# Vitamin D receptor (VDR) on the cell membrane of mouse macrophages participates in the formation of lipopolysaccharide tolerance: mVDR is related to the effect of artesunate to reverse LPS tolerance

**DOI:** 10.1186/s12964-023-01137-w

**Published:** 2023-05-29

**Authors:** Yu Zhang, Jun Zhou, Ling Hua, Pan Li, Jiaqi Wu, Shenglan Shang, Fei Deng, Jing Luo, Mengling Liao, Nuoyan Wang, Xichun Pan, Yue Yuan, Yue Zheng, Yonglin Lu, Yasi Huang, Jiang Zheng, Xin Liu, Xiaoli Li, Hong Zhou

**Affiliations:** 1 Key Laboratory of Basic Pharmacology of Ministry of Education and Joint International Research Laboratory of Ethnomedicine of Ministry of Education, Zunyi Medical University, Zunyi, Guizhou 563000 China; 2 Department of Pharmacology, College of Pharmacy, Army Medical University (Third Military Medical University), Chongqing, 400038 PR China; 3 Medical Research Centre, Southwest Hospital, Third Military Medical University (Army Medical University), Chongqing, 400038 PR China; 4 Department of Pharmacology, College of Pharmacy, Chongqing Medical University, Chongqing, 400016 PR China; 5 Chongqing Key Laboratory of Drug Metabolism, Chongqing, 400016 China

**Keywords:** Membrane vitamin D receptor, LPS tolerance, Artesunate, Lipid raft, Caveolin-1

## Abstract

It is unclear whether membrane vitamin D receptor (mVDR) exists on the macrophage membrane or whether mVDR is associated with lipopolysaccharide (LPS) tolerance. Herein, we report that interfering with caveolae and caveolae-dependent lipid rafts inhibited the formation of LPS tolerance. VDR was detected as co-localized with membrane molecular markers. VDR was detected on the cell membrane and its level was higher in LPS-tolerant cells than that in only LPS treatment cells. Anti-VDR antibodies could abolish the effect of artesunate (AS) to reverse LPS tolerance, and the wild-type peptides (H397 and H305) of VDR, but not the mutant peptide (H397D and H305A), led to the loss of AS’s effect. AS decreased the mVDR level in LPS-tolerant cells. In vivo, AS significantly reduced VDR level in the lung tissue of LPS-tolerant mice. In summary, mVDR exists on the cell membrane of macrophages and is closely associated with the formation of LPS tolerance and the effects of AS.

Video Abstract

**Supplementary Information:**

The online version contains supplementary material available at 10.1186/s12964-023-01137-w.

## Introduction

Sepsis is a life-threatening organ dysfunction syndrome caused by host response disorders related to infection and/or infectious factors. Sepsis can lead to septic shock and multiple organ dysfunction syndrome (MODS), with a mortality rate of 30%—70% [1]. The occurrence of sepsis is closely related to dysfunction of the immune system, in which the innate immune system, such as the monocyte-phagocyte system, as the body's first line of defense, plays an early and important role [2–5]. During the pathophysiological process of sepsis, patients in the intensive care unit (ICU) die more frequently in the immunosuppression phase than in the cytokine storm phase because of decreased clearance of bacteria [1] Therefore, the impaired function of monocytes is closely related to the decreased removal of bacteria.

Lipopolysaccharides (LPS), an outer membrane component of gram-negative bacteria, is a highly potent activator of the innate immune system. It can be recognized by Toll-like receptor 4 (TLR4) and then activates nuclear factor kappa B (NFκB) signaling pathway, inducing the expression of various pro-inflammatory cytokines such as tumor necrosis factor alpha (TNF-α), interleukin (IL)-6 and IL-1β [6]. Repetitive exposure to small amounts of LPS might lead to reprogramming of the immune response such that lower amounts of pro-inflammatory cytokines are produced even when the host is re-exposed to large doses of bacterial components [7, 8]. This process is called LPS tolerance and LPS tolerance models are widely used in the simulation of the sepsis immunosuppression state both in vitro and in vivo [9]. However, the pathophysiological mechanism of LPS tolerance remains unclear.

Vitamin D_3_ receptor (VDR), which recognizes and binds 1 alpha, 25-dihydroxyvitamin D_3_ (1α,25(OH)_2_D_3_), is a member of the steroid hormone/thyroid hormone receptor superfamily. VDR is considered to exist in two forms: cytoplasmic VDR (cVDR) and nuclear (nVDR); but there has been no report of such a receptor existing on the macrophage cell membrane (mVDR). cVDR can translocate into the nucleus, and then nVDR binds preferentially as a heterodimer with the retinoid X receptor (RXR) to hexameric repeats on vitamin D response elements (VDRE) in the promoter regions of target genes, such as *ATG16L1* (encoding autophagy related 16 like 1) [10, 11].

Studies from clinical patients have found that a severe deficiency in serum 1α,25(OH)_2_D_3_, exacerbated by low levels of vitamin D binding protein, is strongly associated with higher mortality from sepsis. These results suggest that 1α,25(OH)_2_D_3_ deficiency is a risk factor for sepsis, at least closely related to sepsis [12]. And 1α,25(OH)_2_D_3_ supplementation in critically ill patients may reduce mortality, and parenteral administration might be associated with a greater impact on mortality [13]. In rats’ sepsis model, 1α,25(OH)_2_D_3_ supplementation has significant effects on coagulation and liver function with reduced thrombocyte count and prothrombin time together with elevated ALT and bilirubin [14]. Since VDR is the receptor for 1α,25(OH)_2_D_3_, 1α,25(OH)_2_D_3_ and VDR are closely related to the occurrence and development of sepsis. Recently we have found that VDR is tightly related to the formation of the formation of LPS tolerance in vitro, and in cecal ligation and puncture (CLP)-induced sepsis immunosuppression, knockdown of VDR led to a loss of the LPS tolerance phenotype [15].

Artesunate (AS) is an effective and reliable anti-malarial drug with low toxicity [16, 17], which possesses several interesting effects, such as anti-inflammation [18–20]. Previously, in our laboratory, we showed that AS protects septic animals by inhibiting pro-inflammatory cytokines release in the cytokine storm stage [18], suggesting that AS could exhibit anti-inflammatory activity during the cytokine storm phase of sepsis. Furthermore, we found AS significantly reduced the mortality of CLP-induced immunosuppression mice challenged with *Pseudomonas Aeruginosa* (PA), and enhanced pro-inflammatory cytokines release and bacterial clearance to reverse sepsis-induced immunosuppression in vivo and in vitro. Mechanistically, AS interacts with VDR, thereby inhibiting the nuclear translocation of VDR, which influences *ATG16L1* transcription and subsequent autophagy activity. In addition, AS inhibited the physical interaction between VDR and NFκB p65 in LPS-tolerant macrophages, and then promoted nuclear translocation of NFκB p65, which activated the transcription of NFκB p65 target genes, including pro-inflammatory cytokines [15].

Although AS is considered to easily cross the cell membrane and can bind to VDR in cytoplasm, thus playing a pharmacological role, how AS acts on VDR is not clear. Occasionally, we found that anti-VDR antibodies abolished the effect of AS to increase the TNF-α release from LPS-tolerant cells without treatment with a permeabilization reagent, suggesting the possible existence of mVDR on the cell membrane. Therefore, in the present study, we aimed to focus on the existence of mVDR on the macrophage membrane, its role during the formation of LPS tolerance, and the importance of mVDR for the effect of AS.

## Materials and methods

### Experimental animals

BALB/c mice and KM mice were used in the experiments. Experiments using specific pathogen free (SPF) grade male BALB/c mice were approved by the Institutional Animal Care and Use Committee of Third Military Medical University (approval number SYXK2017-0002) and were performed in accordance with relevant guidelines. These mice (weighing 18—22 g, 6—8 weeks old) were obtained from Beijing HFK Bioscience Co., LTD (Beijing, China) and housed in a pathogen free environment and fed with free access to food and water. The environment was controlled with the room temperature maintained at 22 ± 2 °C and artificial light–dark cycles of 12 h.

SPF grade KM mice (male, 4—6 weeks old, weighing 18—22 g) were purchased from Hunan SJA Laboratory Animal CO., LTD (Hunan, China). The mice were fed in an individually ventilated cage (IVC) grade animal house of the experimental building of Zunyi Medical University, with free access to feed and water, and were maintained on a 12 h light/dark cycle at 22.0 ± 2.0 °C, with a humidity of 60.0 ± 5.0%. All protocols and experiments procedures involving live animals were approved by the Animal Care Welfare Committee of Zunyi Medical University (approval number SYXK2021-0003).

### Cells and culture

Mouse peritoneal macrophages (PMs) were isolated from male KM mice. The mice were intraperitoneally injected with 3 mL of 3% Thioglycolate (Sigma-Aldrich, St. Louis, MO, USA) on the first day, and the cells were isolated after anesthesia on the third day. The mice were injected with 5 mL of normal saline (NS), gently rubbed for 2—3 min, the peritoneal supernatant was collected, centrifuged, and the supernatant was discarded, and the peritoneal cells were suspended in fresh Dulbecco’s modified Eagle’s medium (DMEM) without fetal bovine serum (FBS) in cell culture dishes at 37 °C in 5% CO_2_.

### Bacterial strain and preparation of bacterial suspension

The PA clinical insolate was kindly provided by Prof. Peiyuan Xia (Southwestern Hospital, Chongqing, China). Bacteria cultured in Mueller–Hinton Broth during the logarithmic phase were collected and diluted in sterile normal saline to achieve a concentration of approximately 1.0 × 10^8^ colony-formation units (CFU)/mL.

### Establishment of the LPS-tolerant mouse model and artesunate treatment

To establish the LPS-tolerance model, mice were randomly divided into two groups (10 mice/group) and injected intraperitoneally with LPS (0.3 mg/kg/day) for 3 days, followed by intravenous injection with LPS (50 mg/kg). The survival rate and body weight of the mice were recorded for 7 days. In another experiment, model mice were anesthetized using isoflurane (Keyuan Pharmaceutical, Shandong, China) inhalation after the last LPS injection. Blood, lung and spleen tissues were collected for analysis.

For AS treatment experiments, LPS-tolerant mice were intramuscularly injected with AS (10 mg/kg) at 0 and 4 h after the last LPS injection. At 12 h after the last LPS challenge, blood, lung and spleen tissues were collected for analysis.

### Establishment of the second hit (bacterial challenge) mouse model and AS treatment

LPS-tolerant mice or normal mice were intraperitoneally injected with PA (the second hit) at 6 h after the last LPS challenge and the survival rate of the mice was observed for 7 days. For AS treatment, LPS-tolerant mice were intraperitoneally injected with the lethal bacterial dose, and AS was injected intramuscularly at 0, 4, and 24 h after the last LPS injection. The survival rate of the mice was observed for 7 days.

To investigate the effect of AS on the bacterial load, LPS-tolerant mice were treated as described and blood, lung, and spleen tissues were collected at 6 h for CFU count assays.

### Establishment of the LPS tolerance cell model

LPS tolerance is used extensively to simulate the sepsis-induced immunosuppression phase in vitro [15]. Herein, a cell model of LPS tolerance was established in mouse PMs. Briefly, cells were cultured with LPS (0111: B4, 5 ng/mL, Sigma-Aldrich) for 4 h. Then, the culture supernatant was replaced followed by the addition of LPS (100 ng/mL) to establish the LPS-tolerance cell model. After an additional 4 h, the culture supernatant was collected, and then TNF-α, as the marker of the formation of the LPS-tolerance model, was assayed.

### The influence of a lipid raft inhibitor on the LPS-tolerance cell model

PMs were pretreated with LPS (5 ng/mL) for 4 h, then incubated with LPS (100 ng/mL) and methyl-β-cyclodextrin (MβCD, 5 mM) (MedChemExpress, Shanghai, China) for an additional 4 h. The supernatant was collected to detect the TNF-α level, which is considered to be a marker for the formation of the LPS-tolerance model.

### The influence of anti-VDR antibodies on the effect of artesunate

Seven anti-VDR antibodies from different manufacturers (Cell Signaling Technology (12550S); Proteintech (67,192–1-IG); Santa Cruz Biotechnology (SC-13133); Boster (BA2877-2); ABclonal Technology Co., Ltd. Wuhan, China (A2194); Abcam (Ab109234), Cambridge, UK; and Bioworld Technology, Minneapolis, MN, USA (BS91492)) were used to observe alterations is AS’s effect to increase the TNF-α release from LPS-tolerant cells. Briefly, PMs were treated with the seven anti-VDR antibodies (anti-VDR antibody: DMEM = 1:100) for 1 h, separately, and then the PMs was pretreated with LPS (5 ng/mL) for 4 h, incubated with LPS (100 ng/mL), with or without AS (injection preparation, Guilin Pharma Corp, Guangxi, People's Republic of China National Medicine Standard H10930195), and anti-VDR antibodies for an additional 4 h. Finally, the supernatants were collected to detect TNF-α level.

### The influence of VDR wild-type and mutant peptides on the effect of artesunate

The two peptide sequences from human VDR (shown in Table 1) containing histidine 397 (wild-type peptide H397) and histidine 305 (wild-type peptide H305) and two mutant peptide sequences with histidine 397 mutated to aspartic acid (mutant peptide H397D) and histidine 305 mutated to alanine (mutant peptide H305A) were synthesized (ChinaPeptides Co., Ltd, Shanghai, China). Briefly, PMs were pretreated with LPS (5 ng/mL) for 4 h, and then AS was added with LPS (100 ng/mL) and the peptides simultaneously. After incubation for another 4 h, the supernatant was collected to detect TNF-α level.

**Table 1:** Wild and mutant polypeptides sequences from human VDR

Polypeptides	Peptide sequence (N → C)
VDR wild peptide (H305)	VSDVTKAG**H**SLELIEPLIKFQVGLK
VDR mutant peptide (H305A)	VSDVTKAG**A**SLELIEPLIKFQVGLK
VDR wild peptide (H397)	LYAKMIQKLADLRSLNEE**H**SKQYRCLS
VDR mutant peptide (H397D)	LYAKMIQKLADLRSLNEE**D**SKQYRCLS

### siRNA transfection in vitro

PMs were transfected with an siRNA targeting *Cav1* (encoding caveolin-1) or a control siRNA using siRNA Transfection Reagent (SANTA) for 6 h. The medium was discarded and the PMs were incubated for a further 24 h with medium supplemented with 20% FBS and 2% antibiotics. The medium was discarded, and the cells were harvested after incubating again for 24 h with medium containing 10% FBS and 1% antibiotics, then the medium was discarded. The PMs were pretreated with LPS (5 ng/mL) for 4 h, then incubated with LPS (100 ng/mL) for an additional 4 h. The supernatant was collected to detect the TNF-α level, and the PMs were collected to detect the level of ATG16L1 using western blotting.

### Molecular docking

The amino acid sequences of human VDR (NP_001017535.1) and mouse VDR (NP_033530.2) were downloaded from GenPept (www.ncbi.nlm.nih.gov/protein), and the crystal structures of the human VDR-1α,25(OH)_2_D_3_ complex (1db1) was obtained from the Protein Data Bank (PDB; www.rcsb.org). The homologous 3D structures of mouse VDR were modelled in the ORCHESTRA program of SYBYL-X2.0 by using the known crystal structures of human VDR as the reference. Molecular docking was carried out using SYBYL-X2.0 employing the Surflex-docking program to investigate the detailed interaction between AS and human VDR. The initial binding pocket of AS was subsequently characterized to be close to HIS397 and HIS305 according to a known crystal structure of the human VDR-1α,25(OH)_2_D_3_ complex [21]. The interactions of human VDR with 1α,25(OH)_2_D_3_ and human VDR with AS, the alignment of different VDRs, and visualizations were performed using an education version of the PyMol package (www.pymol.org).

### Binding assay

To confirm the binding of AS to mVDR, cell membrane proteins were extracted, and then mVDR in the extracts was captured to the 96-well plates coated with anti-VDR antibodies (Wuhan Fine Biotech, Wuhan, China). After another anti-VDR antibody (Cell Signaling Technology, USA) targeting another epitope was added to the plate, FITC-labeled AS (Xi'an Rui Xi Biotechnology Co., Ltd., Xi'an, China) was added to the plate and the fluorescence values was finally tested.

### Immunofluorescence assay

For cell membrane VDR detection, VDR was labeled with green fluorescent fluorescein FITC. The cell membrane was stained with the red fluorescent DIL, vimentin, or an anti-CD64 antibody.

For cytoskeletal membrane dye labeling, PMs were collected to fixed using 4% paraformaldehyde for 1 h at room temperature, then blocking was performed with goat serum for 1 h at room temperature. The PMs were incubated overnight (over 16 h) at 4 °C with anti-VDR antibody (Proteintech, Wuhan, China), followed by incubation with FITC-goat anti-mouse IgG secondary antibodies (Boster, Shanghai, China). The cells were then incubated with DIL (Solarbio), and anti-vimentin antibody (Beyotime Biotechnology, Shanghai, China) for 10 min at room temperature, respectively. Nuclei were stained using 2-(4-amidinopheny l)-1H-indole-6-carboxamidine (DAPI) (Boster) for 10 min at room temperature. Finally, the cells were photographed under a laser confocal microscope (Leica, Heidelberg, Germany) to observe VDR on the cell membrane.

For red fluorescent anti-CD64 antibody labeled cell membranes, after fluorescent labeling of VDR, PMs were again blocked with serum and incubated with the anti-CD64 antibody (Santa Cruz Biotechnology) or anti-CD64 antibody's control antibody mouse IgG (Beyotime Biotechnology) overnight at 4 °C, followed by incubation with Alexa Fluor 594 (594) goat anti-mouse IgG secondary antibody (Boster). Nuclei were stained using DAPI. Finally, the cells were photographed under a laser confocal microscope (Leica) to observe VDR on the cell membrane.

For cytoplasmic and nuclear VDR detection, PMs were fixed with 4% paraformaldehyde for 1 h at room temperature, permeabilized for 10 min in 0.3% Triton X-100 (Solarbio), resuspended in phosphate buffered saline (PBS). Blocking was performed with goat serum for 1 h at room temperature. The PMs were incubated overnight (over 16 h) at 4 °C with anti-VDR antibodies (Proteintech), followed by incubation with FITC sheep anti-rabbit IgG secondary antibodies (Boster). The nuclei were stained using DAPI (Boster) for 10 min at room temperature. Finally, the cells were photographed under a laser confocal microscope (Leica) for the expression of molecules in the cytosol and nuclei.

### Immunohistochemistry

Sections were deparaffinized and hydrated, and then heat-induced epitope retrieval was performed with Citrate Antigen Retrieval Solution (pH 6.0) for 15 min at 100 °C. The sections were peroxide blocked for 10 min, then blocking was performed with goat serum for 1 h at room temperature, then incubated overnight (over 16 h) at 4 °C with anti-VDR antibody (SANTA), followed by incubation with Enhanced enzyme-labeled goat anti-mouse IgG polymer (Zsbio, Beijing, China) for 30 min, Then further treated with the Biotin-Streptavidin HRP Detection Systems (Zsbio) for 15 min, DAB for 10 min, and a hematoxylin counterstain for 5 min. Sections were dehydrated through a series of ascendingethanol concentrations and xylene, neutral gum mounting, finally, photographed under a light microscope (Olympus, Tokyo, Japan).

### Extraction of membranal, cytoplasmic and nuclear proteins

Membrane proteins were extracted from PMs using a cell membrane protein extraction kit (Thermo Fisher Scientific, Waltham, MA, USA). Cytoplasmic and nuclear proteins from PMs were extracted using a nucleoprotein Extraction Kit (Solarbio).

### Western Blotting

Total cellular protein from PMs was extracted using radioimmunoprecipitation assay (RIPA) buffer (Cell Signaling Technology). The proteins were quantified using a BCA Protein Quantification Kit (GENEray, Shanghai, China). Ten micrograms of each protein sample were separated using sodium dodecyl sulfate polyacrylamide gel electrophoresis (SDS-PAGE) and transferred to a polyvinylidene fluoride (PVDF) membrane. The membranes were blocked with 5% skim milk and incubated with different antibodies [anti-VDR (Santa Cruz Biotechnology, Dallas, TX, USA), anti-ATG16L1 (Cell Signaling Technology)] overnight (over 16 h) at 4 °C. The membrane was washed three times for 10 min each time using Tris-buffered saline containing 0.1% Tween 20 (TBST), incubated with the corresponding secondary antibodies, and washed again. Immunoreactive protein bands were imaged using a chemiluminescent gel imaging system (Bio-Rad, Hercules, CA, USA) and analyzed using Image Lab software (Bio-Rad).

### Enzyme-linked immunosorbent assay (ELISA)

Serum, cell culture supernatants and tissue homogenates were collected and the levels of TNF-α, IL-6 and IL-1β were detected using respective ELISA kits (Thermo Fisher Scientific). The VDR protein level was detected using a VDR ELISA Kit (JiangLai Biology, Shanghai, China; Wuhan Fine Biotech, Wuhan, China).

### Statistical analysis

All experiments were repeated more than three times, and all values are presented as the mean ± standard deviation (SD). Statistical analysis was performed and plotted using one-way analysis of variance (ANOVA) followed by two tailed unpaired Student's *t*-test using GraphPad prism 8.0.2 software (GraphPad Inc. La Jolla, CA, USA). In the figures, *, *P* < 0.05, significant statistical difference; **, *P* < 0.01, significant statistical difference; ^#^, *P* > 0.05, no statistical difference.

## Results

### Interfering with the internalization pathway inhibits the formation of LPS tolerance

**Fig. 1:**
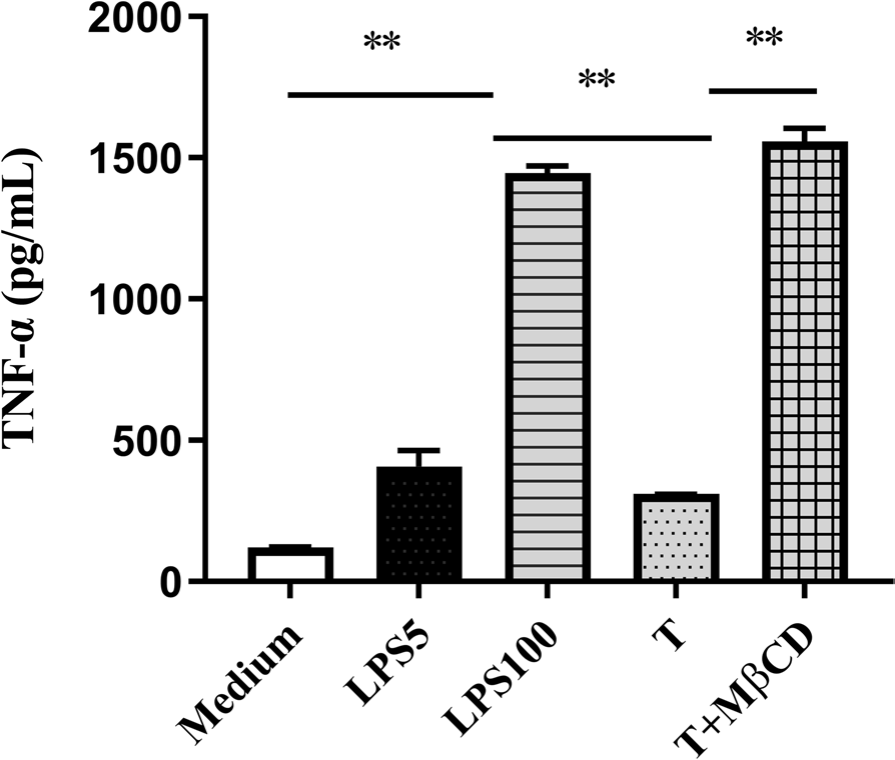
The effect of MβCD on TNF-α release in LPS-tolerant cells. PMs were pretreated with 5 ng/mL LPS (LPS5) for 4 h, and then incubated with 100 ng/mL of LPS (LPS100) and 5 mM of MβCD for an additional 4 h. The supernatant was collected to detect the TNF-α level (*n* = 3). *, *P* < 0.05; **,* P* < 0.01. MβCD: methyl-β-cyclodextrin; LPS5: 5 ng/mL LPS; LPS100: 100 ng/mL LPS (LPS100); T: LPS tolerance

Previous studies reported that 1α,25(OH)_2_D_3_ can bind protein disulfide isomerase family A member 3 (PDIA3), a receptor on the cell membrane, and is then internalized into cells through the lipid raft pathway [22]. Our previous results showed that VDR is closely related to the formation of LPS tolerance [15]. Therefore, we wondered whether the lipid raft internalization pathway is related to the formation of LPS tolerance. Herein, a cholesterol-depleting agent (MβCD), as a lipid raft inhibitor, was used to destroy the lipid rafts to observe the formation of the LPS tolerance. The results showed that LPS-tolerant cells (PMs were pretreated with LPS at 5 ng/mL followed by treatment with LPS at 100 ng/mL) released less TNF-α than LPS (100 ng/mL) only-treated cells (hereafter referred to as non-tolerant cells). However, MβCD significantly increased TNF-α release from LPS-tolerant cells compared with that from non-tolerant cells (Fig. 1), suggesting that MβCD destroyed the lipid rafts, which then inhibited the formation of LPS tolerance.

### Inhibition Caveolin’s function and expression blocks the formation of LPS tolerance

**Fig. 2:**
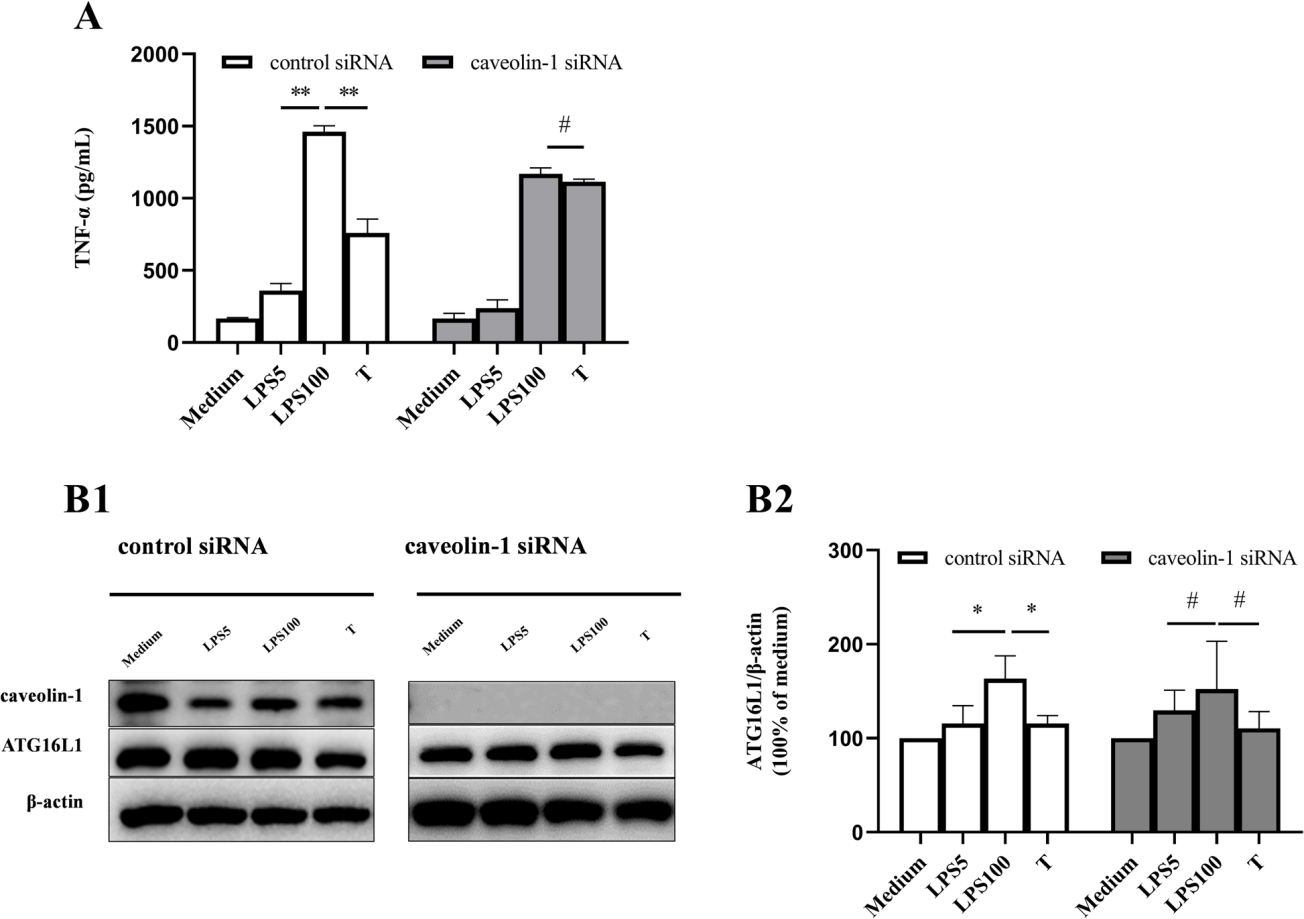
The effect of caveolin-1 siRNA on the formation of the LPS-tolerant cell model. **A** The effect of caveolin-1 siRNA on TNF-α level in LPS-tolerant cells (*n *= 3). **B1**, **B2** The effect of caveolin-1 siRNA on the ATG16L1 protein level in LPS-tolerant cells (*n* = 3). *, *P* < 0.05; **, *P* < 0.01; ^**#**^, *P* > 0.05. LPS5: 5 ng/mL LPS; LPS100: 100 ng/mL LPS (LPS100); T: LPS tolerance; ATG16L1, autophagy related 16 Like 1

Caveolin-1 is a key molecule that ensures the integrity and function of lipid rafts [23] and 1,25(OH)_2_D_3_ can internalize into cells via caveolin-dependent endocytosis [24, 25]. To further clarify whether the formation of LPS tolerance is related to caveolin-dependent lipid raft pathway, PMs were transfected with a siRNA targeting *Cav1* (encoding caveolin-1) (Fig. 2A, B1). The results showed there was no difference in TNF-α level between the non-tolerant cells and the LPS-tolerant cells transfected with a siRNA targeting *Cav1* (Fig. 2A), meanwhile there was also no difference in the ATG16L1 level between two treatment groups (Fig. 2B1, B2), demonstrating that caveolin-dependent internalization pathway is tightly related to the formation of LPS tolerance.

### VDR exists on the cell membrane and membrane VDR is related to the formation of LPS tolerance

**Fig. 3:**
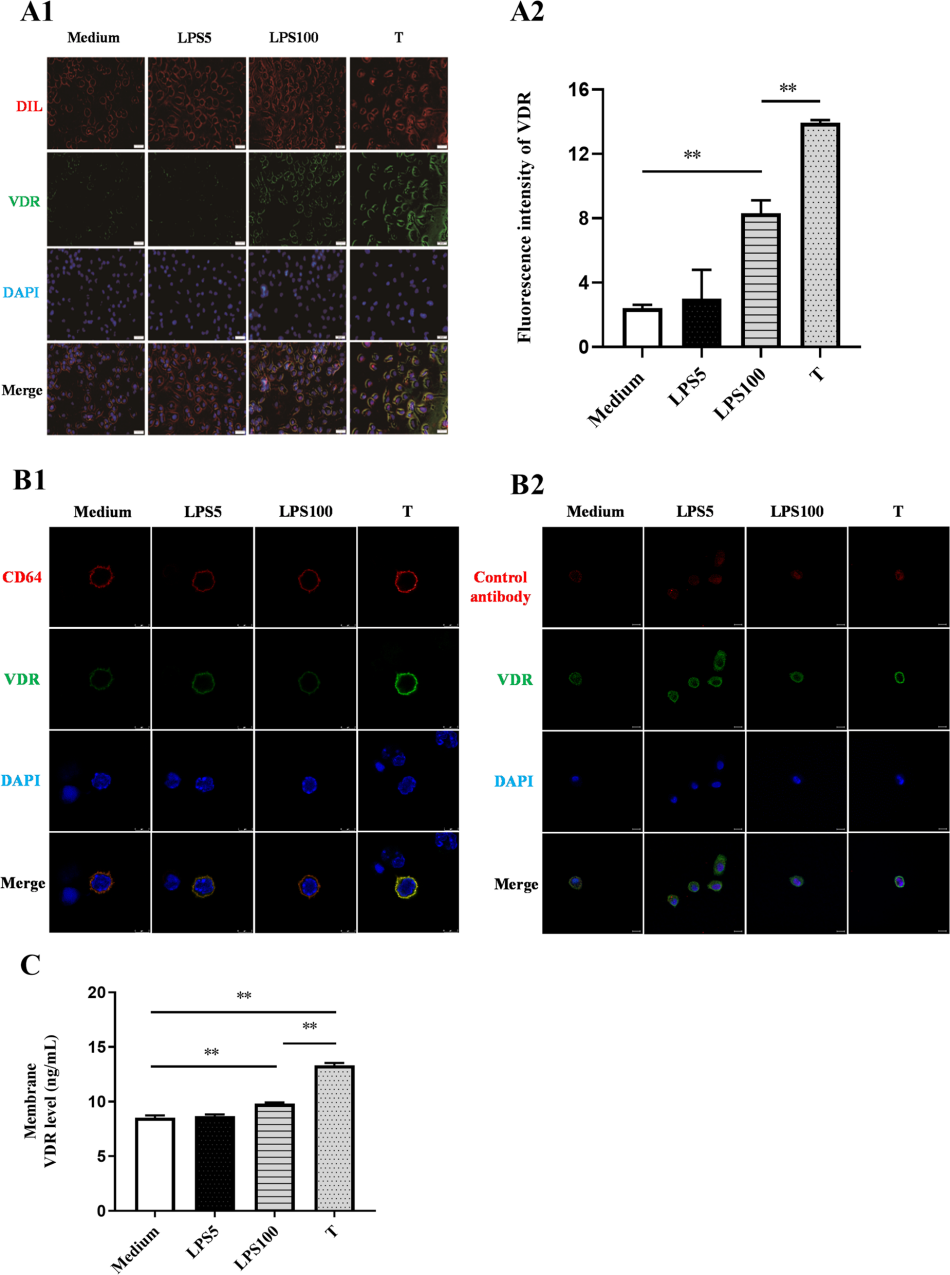
The detection of mVDR in LPS-tolerant cells. **A** Co-localization of VDR and DIL in LPS-tolerant cells (*n* = 3). mVDR was labeled green with the anti-VDR antibody, the cell membrane was labeled red with DIL (**A1**), and the fluorescence intensities were calculated (**A2**). **B** Co-localization of VDR and CD64 in LPS-tolerant cells (*n* = 3). mVDR was labeled green with the anti-VDR antibody, the cell membrane was labeled red with CD64 (**B1**), and mVDR was labeled green with the anti-VDR antibody, the homologous control antibody was labeled red with mouse IgG (**B2**). **C** Membrane VDR level in LPS-tolerant cells (*n* = 3). **, *P* < 0.01; ^#^, *P* > 0.05. LPS5: 5 ng/mL LPS; LPS100: 100 ng/mL LPS (LPS100); T: LPS tolerance; DIL, 1,1'-dioctadecyl-3,3,3',3'-tetramethylindocarbocyanine perchlorate. mVDR: membrane VDR

Previously, 1α,25(OH)_2_D_3_ was found to be internalized into cells through caveolin-mediated pathway by binding to PDIA3 [26]. However, it is unclear whether VDR exists on the cell membrane (mVDR), and whether mVDR is involved in the formation of LPS tolerance. Therefore, 1,1'-dioctadecyl-3,3,3',3'-tetramethylindocarbocyanine perchlorate (DIL), a membrane non-specific marker, was firstly used to label the cell membrane of mouse peritoneal macrophages (red fluorescence); and VDR was labeled with an antibody fused with green fluorescent fluorescein isothiocyanate (FITC). The results showed that the green fluorescence was obviously increased in LPS-tolerant cells compared with that in non-tolerant cells, suggesting that VDR level was higher in LPS-tolerant cells. More importantly, the green and red fluorescence were co-located in LPS-tolerant cells, without treatment with permeabilization reagent (in which case the antibody could not enter the cell) (Fig. 3A1, A2), suggesting that VDR was present on the cell membrane.

Secondly, the cell membrane was labeled with CD64 (a marker of the macrophage membrane) with red fluorescence and VDR was labeled using the antibody with FITC green fluorescence. The results showed the green fluorescence was obviously increased in LPS-tolerant cells compared with that on non-tolerant cells. Most importantly, there was co-localization of green and red fluorescence (Fig. 3B1). However, isotype IgG antibody, a homologous control antibody of CD64 antibody was used to observe whether the control antibody and VDR were co-located. The results showed that there was no co-localization between VDR and control antibodies (Fig. 3B2). These results indicated that red fluorescently labeled CD64 antibodies bind to the CD64 receptor on the macrophage surface and co-localization with green fluorescently labeled VDR on the cell membrane, again suggesting that VDR exists on the cell membrane.

To further confirm that VDR is present on the membrane, cell membrane proteins were extracted and the level of VDR of the extracted protein was tested using a VDR ELISA kit. The results showed that VDR was detected in the extracted cell membrane proteins, demonstrating that VDR exists on the cell membrane. Meanwhile, the level of VDR was higher among the extracted protein from LPS-tolerant cells than in the proteins extracted from the non-tolerant cells **(**Fig. 3C**)**. Therefore, the above results strongly demonstrated that VDR exists on the cell membrane and mVDR level was markedly increased in the LPS tolerance cells.

### Artesunate’s effect to reverse the formation of LPS tolerance is tightly related to its binding to mVDR

Our previous reports found that AS can reverse the formation of LPS tolerance in vitro and protected CLP-induced immunosuppressed mice [15, 27]; therefore, is the effect of AS related to mVDR?

**Fig. 4:**
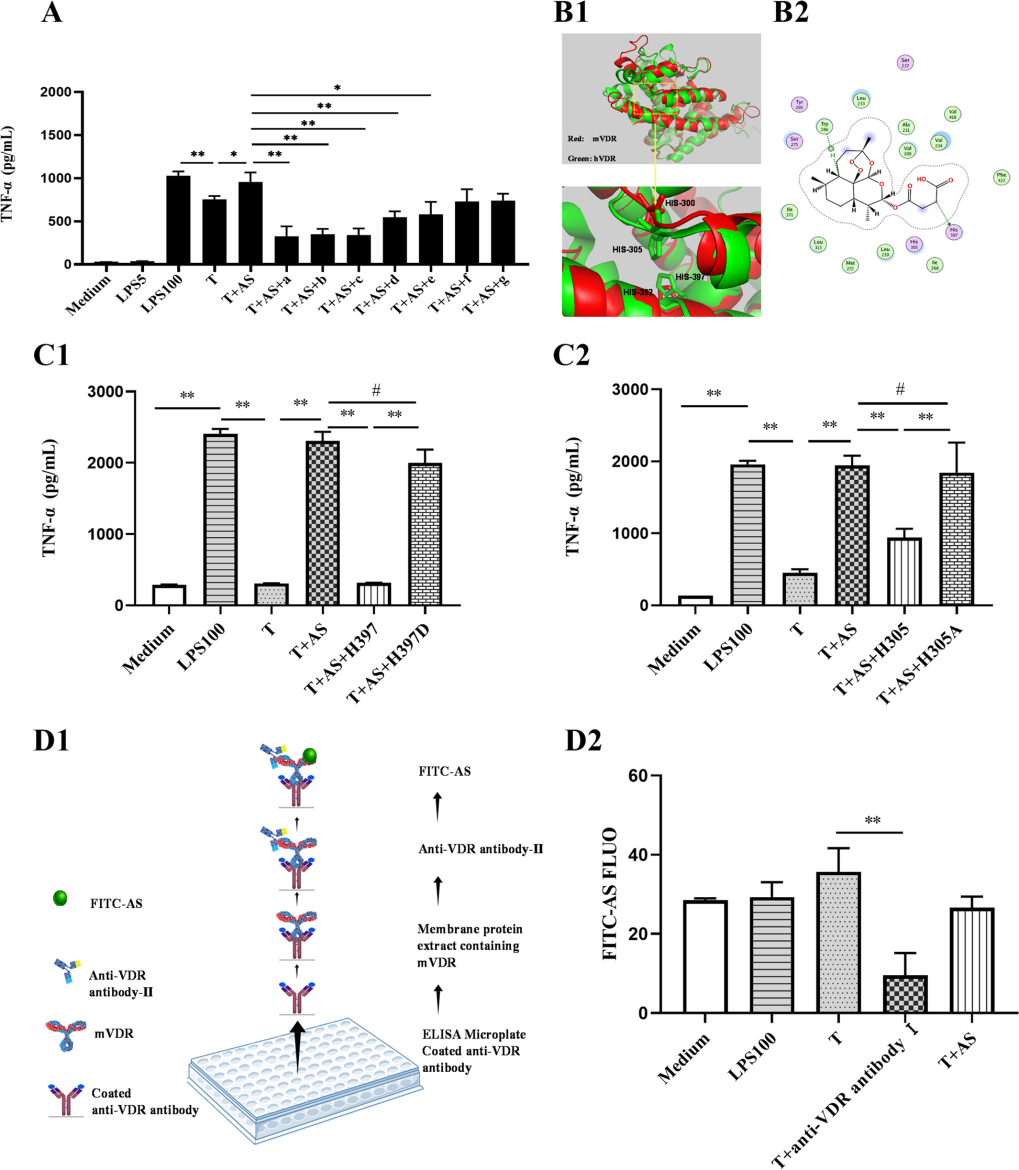
Anti-VDR antibodies abolish the effect of artesunate. **A** The effect of seven anti-VDR antibodies from different manufacturers on artesunate (AS)-mediated increase in TNF-α levels in LPS-tolerant cells (*n* = 3). Note: a—g represent the anti-VDR antibodies from CST, Boster, Proteintech, Santa Cruz Biotechnology, ABclonal, Abcam, and Bioworld Technology, respectively. **B** Molecular docking. Human VDR (green) and mouse VDR (red) have similar spatial structures (**B1**). Histidine 305 and 397 of human VDR (equivalent to histidine 300 and 392 of mouse VDR) are important for VDR binding to AS (**B2**). **C** Effect of peptides on the AS-mediated TNF-α increase in LPS-tolerant cells (*n* = 3). The peptides are the peptide H397 and its mutated peptide H397D (**C1**) or the peptide H305 and its mutated peptide H305A (**C2**). **D** Illustration of the binding of mVDR and AS by ELISA. Schematic diagram of the binding assay (**D1**). Effect of anti-VDR on the binding of AS and VDR tracked by FITC-AS (*n* = 3). AS with fluorophore Fluorescein 5-isothiocyanate was named FITC-AS (D2). *, *P* < 0.05; **, *P* < 0.01; ^**#**^, *P* > 0.05. LPS5: 5 ng/mL LPS; LPS100: 100 ng/mL LPS (LPS100); T: LPS tolerance

Antibodies from different companies probably target different antigenic determinants of VDR; therefore, seven antibodies from seven different companies were used. The results showed five of the seven anti-VDR antibodies could abolish the effect of AS to increase TNF-α release from LPS-tolerant cells (Fig. 4A), without treatment using a permeabilization reagent, which suggested that mVDR was associated with AS’s effect to reverse the formation of LPS tolerance.

These results suggested that AS might interact with VDR, but where is the site of interaction? Herein, a molecular docking simulation was firstly carried out to predict the site of VDR binding to AS. The results showed that the spatial structure of mouse VDR is similar to that of human VDR (Fig. 4B1, B2), and the binding site of human VDR to AS is also located around H397 and H305 (Fig. 4B3).

Based on above prediction, the wild-type peptides with H397 and H305 (H397 and H305) of VDR and the mutant peptide (H397D and H305A) were synthesized. Based on the theory that AS could bind (H397 and H305, but cannot bind H397D and H305A), if AS is preincubated with H397 and H305, then the mixture is added into the cell culture system in which there is insufficient AS molecules to bind mVDR, the effect of AS to reverse the formation of LPS tolerance will reduce or even abrogated. In contrast, if AS is preincubated with H397D and H305A, and the mixture is added into the cell culture system in which there is sufficient AS molecules to bind to mVDR, the effect of AS to reverse the LPS-tolerance cell model would persist. Herein, the results showed that preincubation of AS and H397 (Fig. 4C1) or H305 (Fig. 4C2) eliminated the effect of AS, manifested as a lower TNF-α level. Meanwhile, preincubation of AS and H397D or H305A did not change the effect of AS (Fig. 4C1, C2), which was manifested as a higher TNF-α level. These results demonstrated that AS indeed binds mVDR, and H397 and H305 of VDR play important roles in AS binding to VDR, suggesting that AS binds to mVDR at least through two binding sites around H397 and H305.

To further verify the interaction between AS and mVDR, an indirect competitive binding assay using FITC-labeled AS was designed (Fig. 4D1). The results showed the FITC fluorescence value was higher than that in Medium and LPS100 because there were lots of mVDR protein molecules in LPS-tolerant cells; but it was lower in LPS-tolerant cells treated with anti-VDR antibody (T + anti-VDR antibody) or AS (T + AS) because the second anti-VDR antibody and pre-treatment of AS occupied the amino acid sites that could bind to FITC-AS (Fig. 4D2). Above results further demonstrated there was mVDR on the membrane of macrophages and AS’s effect was closely tightly related to its binding to mVDR.

### AS decreases the levels of mVDR, cVDR, and nVDR in LPS-tolerant cells

**Fig. 5:**
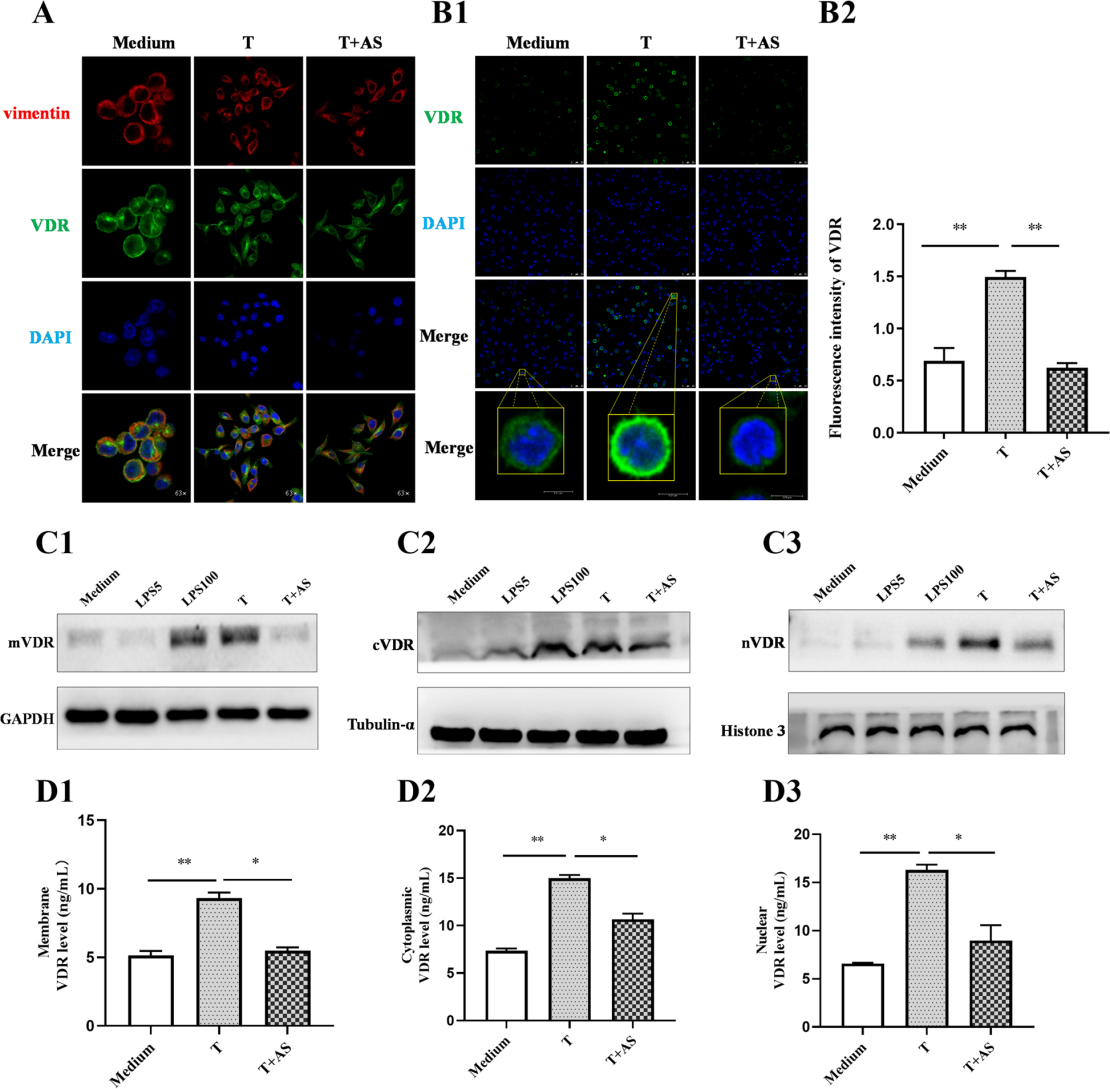
Artesunate affects the level of membrane, cytoplasmic, and nuclear VDR in LPS-tolerant cells. **A** The effect of artesunate (AS) on the mVDR level in LPS-tolerant cells without a permeabilization reagent treatment under laser confocal microscopy (*n* = 3). mVDR was labeled green with the anti-VDR antibody, the cell membrane was labeled red with vimentin. **B** The effect of AS on the VDR level in LPS-tolerant cells with a permeabilization reagent treatment under laser confocal microscopy (*n* = 3). cVDR was labeled green with the anti-VDR antibody **(B1)**, and the fluorescence intensities were calculated (**B2**). **C** Detection of mVDR (**C1**), cVDR (**C2**), and nVDR (**C3**) levels by Western blotting. **D** Detection of mVDR (**D1**), cVDR (**D2**), and nVDR (**D3**) levels by ELISA, respectively. *, *P* < 0.05; **, *P* < 0.01. LPS5: 5 ng/mL LPS; LPS100: 100 ng/mL LPS (LPS100); T: LPS tolerance

VDR is an important nuclear transcription factor, and needs to be transferred from the cytoplasm into nucleus, where it exerts its role in regulating the transcription of target genes [28]. Our previous results showed that in LPS-tolerant cells VDR could be translocated from the cytoplasm to the nucleus in large quantities, and AS can significantly inhibit the nuclear translocation of VDR [15].The above results showed that AS could bind to mVDR; therefore, can AS further reduce the contents of VDR in the cytoplasm and the nucleus by decreasing the internalization of mVDR into the cytoplasm? Therefore, the influence of AS on VDR on the cell membrane, cytoplasm, and nucleus was observed using confocal laser microscopy. In the experiment, the cytoskeleton was labeled with red fluorescent vimentin antibody to show the edge of cell without treatment with a permeabilization reagent, and VDR was labeled with green fluorescence. The results showed the green fluorescence located on the edge of cell; there was less green fluorescence on the edge of the cell without any treatment, but the fluorescence increased in LPS-tolerant cells and decreased in AS-treated LPS-tolerant cells (Fig. 5A), suggesting that AS could decrease the mVDR level in LPS-tolerant cells. Furthermore, our results showed that in LPS-tolerant cells, green fluorescence was distributed in the cytoplasm and nucleus, but more prominently in the cytoplasm. However, AS could significantly change the distribution of green fluorescence, with significantly reduced green fluorescence in the cytoplasm, and almost no green fluorescence in the nucleus (Fig. 5B), demonstrating that AS reduced the content of cVDR and also inhibited the nuclear translocation of cVDR. Combining the above two results, AS was considered to reduce the content of cVDR in the cytoplasm by inhibiting the internalization of mVDR and reducing the nuclear translocation of cVDR, leading to a reduction in the nVDR content.

To further confirm the effect of AS on VDR, cell membrane proteins, cytoplasmic proteins, and nuclear proteins were extracted, respectively; and the levels of mVDR, cVDR and nVDR were determined using two methods of western blotting and ELISA. The results from Western blotting showed that the levels of mVDR, cVDR and nVDR were higher in LPS-tolerant cells than those in non-tolerant cells, but AS markedly decreased the levels of mVDR, cVDR and nVDR in LPS-tolerant cells (Fig. 5C1—C3). The result from ELISA is consistent with those from Western blotting method (Fig. 5D1, D3), further supporting the view that AS inhibited mVDR internalization and then decreased the cVDR level and its subsequent nuclear translocation.

### AS reverses the LPS-tolerant state in LPS-tolerant mice via inhibition mVDR level in vivo and increases pro-inflammatory cytokine levels and decreases bacterial loads in LPS-tolerant mice

**Fig. 6:**
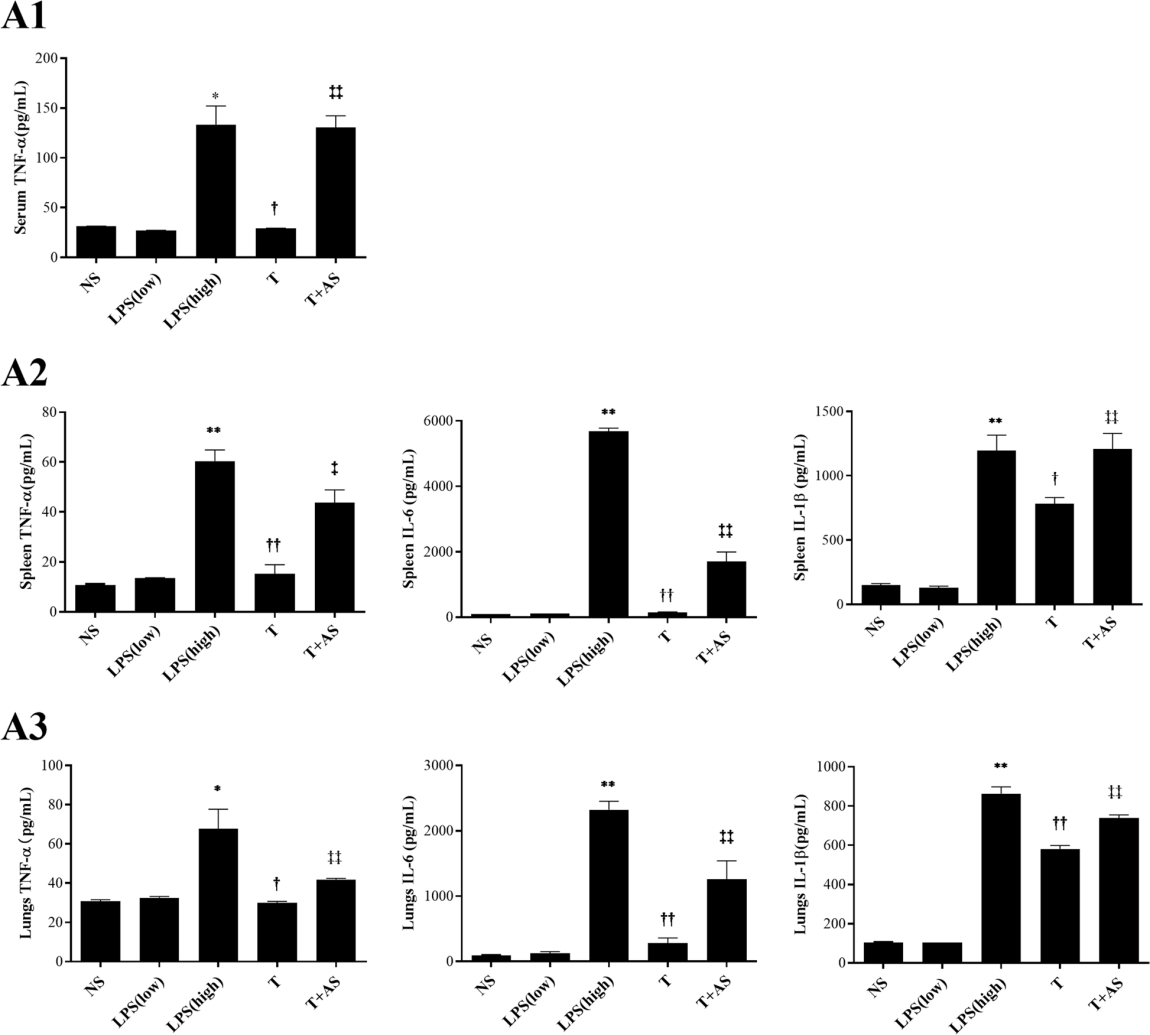
Effects of artesunate on TNF-α, IL-6 and IL-1β levels in an LPS-tolerance mouse model. TNF-α, IL-6, and IL-1β levels of serum (**A1**), spleen (**A2**), and lung (**A3**) were detected in the LPS-tolerant mouse model after artesunate (AS) treatment using ELISA kits, respectively (*n* = 6). *, *P* < 0.05 and **,* P* < 0.01 *vs*. NS; ^†^, *P* < 0.05 and ^††^,* P* < 0.01 *vs*. LPS (high); ^‡^, *P* < 0.05 and ^‡‡^, *P* < 0.01 *vs*. T. LPS(low): 0.3 mg/kg/day LPS for 3 days; LPS(high):50 mg/kg LPS; T: LPS tolerance

The LPS-tolerant mouse model is a classical animal model in addition to the CLP-induced sepsis immunosuppression model. Previously, the CLP-induced sepsis immunosuppression model was established and the effect of AS was observed [15]. However, the effect of AS on the LPS-tolerant mouse model remains unclear. To further determine the effect of AS in reversing the immunosuppressive state of sepsis in vivo, the LPS-tolerant mouse model was established and then pro-inflammatory cytokine levels were tested. The results showed that the serum TNF-α level was much lower in the LPS-tolerant mice than in the non-tolerant mice (Fig. 6A1). However, AS (10 mg/kg) markedly increased the level of TNF-α. For pro-inflammatory cytokines (TNF-α, IL-6 and IL-1β) levels in the spleen and lungs, AS increased the levels of the three pro-inflammatory cytokines, as well as serum TNF-α level (Fig. 6A2, A3), demonstrating that AS could reverse the LPS-tolerant state.

**Fig. 7:**
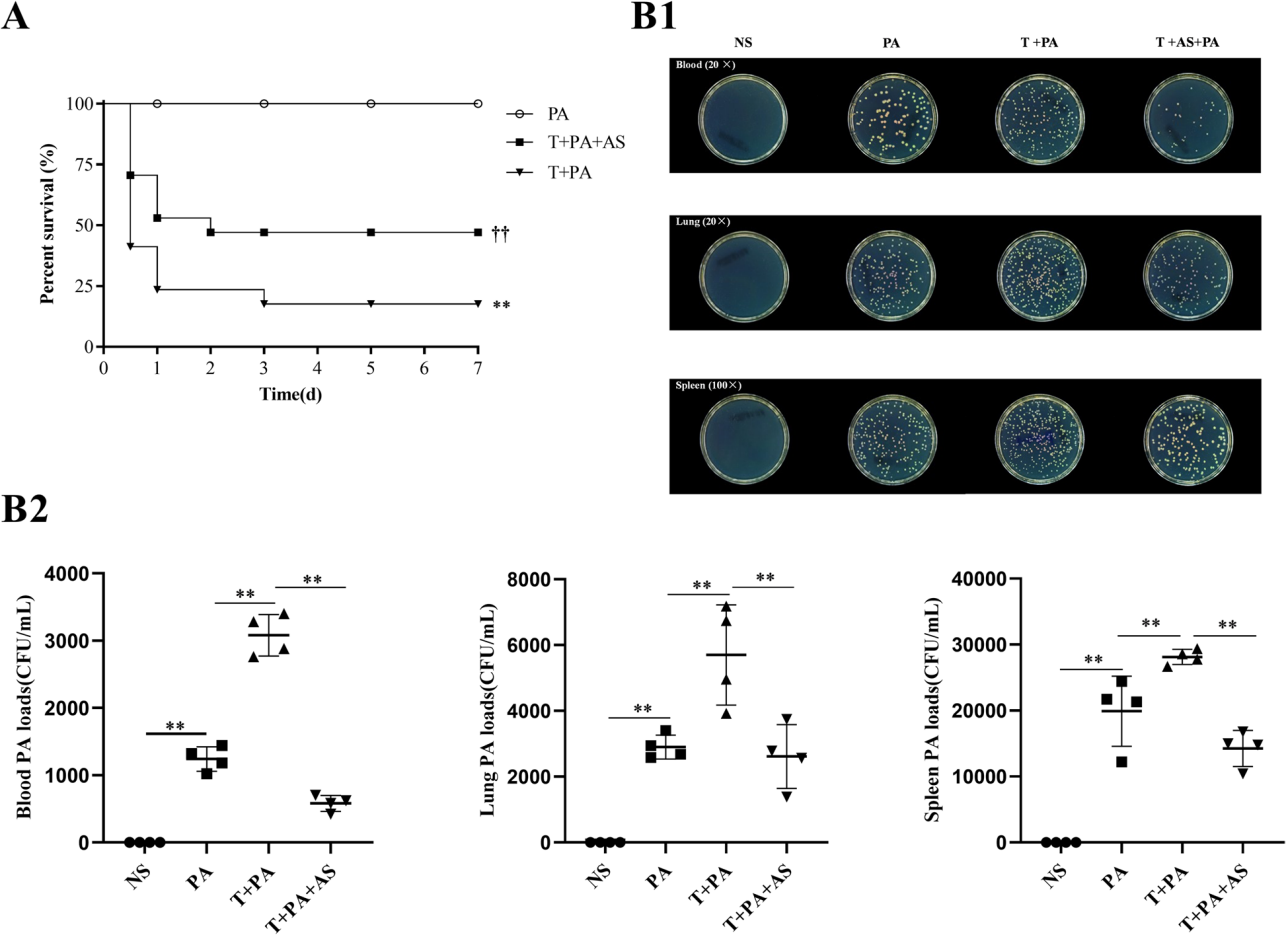
Protection of artesunate on the LPS-tolerant mouse model accepting a second bacterial hit. **A** Effect of artesunate (AS) on the survival of mice in LPS-tolerant mouse model accepting a second bacterial hit. **, *P* < 0.01 *vs*. PA; ^††^, *P* < 0.01 *vs*. T + PA. **B** Effect of AS on bacterial loads of the blood, spleen, and lung of LPS-tolerant mice challenged with *Pseudomonas Aeruginosa* (*n* = 3). Bacteria growing on flat plates (**B1**) and bacterial count analysis results (**B2**). **, *P* < 0.01; PA: Normal mice were only challenged with *Pseudomonas Aeruginosa*; T + PA: LPS tolerance mice were challenged with *Pseudomonas Aeruginosa*; T + PA + AS: LPS tolerance mice were challenged with *Pseudomonas Aeruginosa* and then accepted with AS treatment

To further evaluate the effect of AS in the LPS-tolerant mouse model after the second bacterial hit; both mouse mortality and the bacterial load were evaluated. In the non-tolerant mice, PA alone did not induce mouse death; however, this dose of PA could obviously induce the death of LPS-tolerant mice (Fig. 7A). Significantly, AS (10 mg/kg) markedly decreased mouse mortality (Fig. 7A), demonstrating that AS could significantly protect LPS-tolerant mice. For the bacterial loads, in the blood, spleen, and lungs tissue, the bacterial load in the LPS-tolerant mice was significantly increased compared with that in the non-tolerant mice, indicating that the LPS-tolerant mice had a reduced ability to eliminate bacteria. However, AS could significantly decrease the bacterial load (Fig. 7B1, B2), demonstrating that AS could improve the ability of LPS-tolerant mice to eliminate bacteria.

### AS decreases VDR level in lung tissues of LPS-tolerant mice

**Fig. 8:**
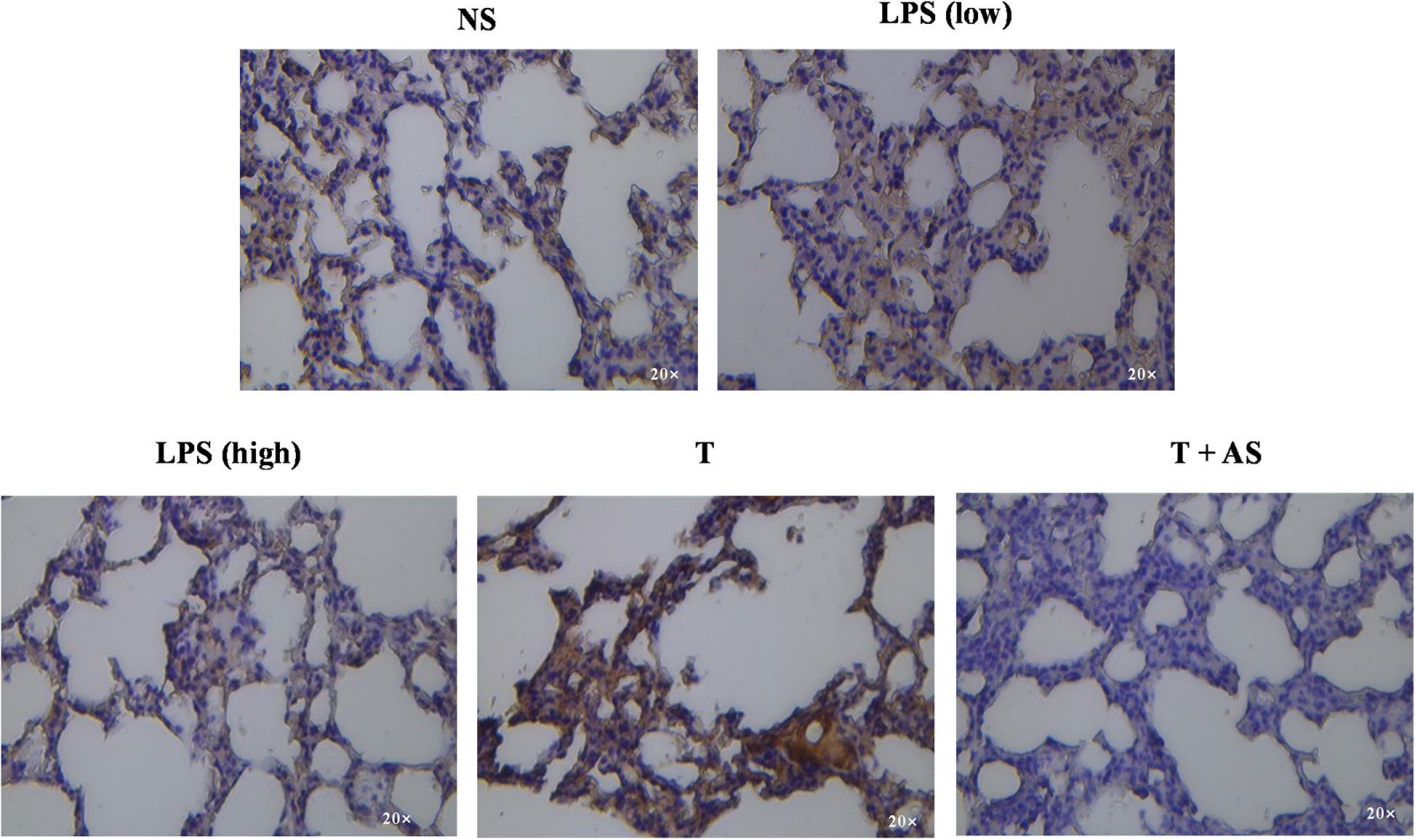
The change of VDR after artesunate treatment. VDR of lung tissue in the LPS-tolerant mouse model after artesunate (AS) treatment was detected using immunohistochemical method. VDR is stained brown. LPS (low): 0.3 mg/kg/day LPS for 3 days; LPS (high): 50 mg/kg LPS; T: LPS tolerance

In the in vitro experiments, the effect of AS was found to be closely related to a reduction in the mVDR level and inhibition of its internalization; however, it was unclear whether the effect of AS was related to the above effects in vivo. Therefore, lung tissue with the most significant injury in the sepsis model was selected and immunohistochemical method was used to observe the changes of VDR level after AS treatment. The results showed that the VDR level (brown) was obviously increased in lungs of LPS-tolerant mice compared to that in non-tolerant mice. However, AS could obviously decrease the VDR level compared with that in the LPS-tolerant mice (Fig. 8), which further demonstrated AS could decrease the VDR level, which was consistent with trends from the in vitro experiments.

## Discussion

This is the first report that mVDR exists on macrophage membrane, and that it is closely related to the formation of LPS tolerance. The protection of AS on CLP-induced sepsis immunosuppression and the formation of LPS tolerance are closely related to the inhibition of mVDR internalization and the reduction of cVDR entry into the nucleus.

Lipid rafts are microdomains on the plasma membrane that are rich in cholesterol and sphingolipids. Lipid rafts are loaded with biologically functional membrane proteins and can promote the internalization of membrane receptor proteins. Previously, it was reported that disrupting lipid raft integrity alters LPS-induced cytokine release [29], demonstrating that LPS-induced inflammation is tightly related to lipid rafts. Herein, MβCD, as a lipid raft inhibitor, inhibited the formation of LPS tolerance, suggesting that lipid rafts are associated with the formation of LPS tolerance.

Caveolae are specialized lipid rafts that serve as signaling pathway platforms, with a characteristic invaginated topology, and a membrane leaflet enriched in sphingolipids and cholesterol. Caveolae can also bud from the plasma membrane into the cell, generally fusing with the early endosome, before recycling back to the plasma membrane [30]. Caveolins are the main structural components of caveolae and also are members of the scaffolding cytosolic proteins; deletion of caveolin can lead to the loss of lipid raft function and disordered cellular function [31, 32]. Among them, caveolin-1 is a key molecule that ensures the integrity and function of lipid rafts. Disrupting caveolin-1 expression would affect the internalization of lipid rafts [23]. Herein, the results showed that siRNA targeting *Cav1* inhibited the formation of LPS tolerance and the expression of the autophagy-related molecule ATG16L1, suggesting that the caveolin-dependent lipid raft pathway is tightly related to the formation of LPS tolerance.

VDR belongs to the steroid hormone/thyroid hormone receptor superfamily; along with 12 receptors such as the glucocorticoid receptor (GR), they are classical endocrine receptors [33]. Although GR has been found in three sites: the cell membrane, cytoplasm, and nucleus, VDR was previously considered to be only present in two sites: the cytoplasm (cVDR) and nucleus (nVDR). Previous studies found mVDR on chicken skeletal muscle cells, chondrocytes, and osteoblasts [34, 35]; however, the existence of mVDR on cell membranes is highly controversial. Notably, there has been no report of mVDR on macrophage membranes, although 1α,25(OH)_2_D_3_ can inhibit LPS-induced release of pro-inflammatory cytokines from macrophages [36]. PDIA3 has been found to bind 1α,25(OH)_2_D_3_ on the cell membrane and can be internalized into cell via a caveolin-dependent lipid raft pathway [37], but PDIA3, unlike VDR, is not a real classical receptor to bind 1α,25(OH)_2_D_3_. Herein, a variety of experimental methods were used to discover and confirm that VDR exists on the cell membrane of PMs and is closely related to the formation of LPS tolerance. Firstly, DIL, a membrane non-specific marker, was used to label the membrane and the results showed co-localization of DIL and VDR. Secondly, an anti-CD64 antibody was used to show the PM cell membrane, and the results showed co-localization of CD64 and VDR. Lastly, the strongest evidence was the presence of mVDR in the extracted cell membrane proteins from macrophages, as assessed using western blotting and ELISA methods, which was in line with the observation from confocal laser microscopy. Therefore, our results strongly demonstrate that mVDR exists on the cell membrane of PMs, which has not been reported in previous studies.

AS is an antimalarial recommended by the World Health Organization (WHO) that possess many important effects, such as anti-inflammation and anti-tumor effects [18, 38–40]. Recently, AS was observed to play an immune-regulating effect in CLP-induced immune-suppression mice in our lab, which is tightly related to its inhibition of both VDR expression and VDR binding to NFκB p65. AS interacted with VDR to prevent the nuclear translocation of VDR and decrease its negative regulation of autophagy related target genes such as *ATG16L1*, and then increase autophagy activity. Additionally, AS interacted with VDR to prevent the interaction between VDR and NF-κB p65, increase the nuclear translocation of NF-κB p65, then augment the transcription of its target genes such as pro-inflammatory cytokines. Above two ways lead to an increase in the bacteria clearance and the pro-inflammatory cytokine releases of macrophages [15]. Our previous results showed that cVDR could significantly transfer into the nucleus, while AS could inhibit such a nuclear translocation, leading to a significant reduction of nVDR in the nucleus [15, 27]. However, it is not clear what role mVDR plays in AS's ability to change the distribution of VDR in cells. Herein, in the absence of a permeabilization reagent, anti-VDR antibodies were firstly to use to block mVDR on the membrane, and the results showed that five of seven VDR antibodies could abolish the effect of AS to increase the TNF-α release from LPS-tolerant cells, suggesting that mVDR is located on cell membrane and mVDR is tightly related to AS’s effect.

Human VDR consists of 427 amino acids, which can be divided into six functional regions: A, B, C, D, E, and F, from the amino terminus to the carboxyl terminus. Each functional region acts differently but cooperates with each other. Region E is the ligand binding region, encoded by *VDR* gene exons V—IX, and is the main site of VDR binding to 1α,25(OH)_2_D_3_ [41, 42]. Our molecular docking analysis showed that the potential exists for AS to bind to human and mouse VDR, predicting that the strength of docking is moderately high, and the binding site might be located around histidine 397 and 305 histidine (H397 and H305) of VDR, which is also important for VDR binding to 1α,25(OH)_2_D_3_ [43, 44]. To confirm this prediction, two peptides containing wild-type H397 and H305 of human VDR, and two mutant peptides (H397D and H305A) were synthesized. The results of competitive binding assays further showed that AS can bind mVDR; and after the binding of AS and VDR, AS lost its effect to increase the TNF-α release from LPS-tolerant cells. These results again demonstrated the presence of mVDR on the cell membrane and that mVDR is tightly related to AS’s effect.

**Fig. 9:**
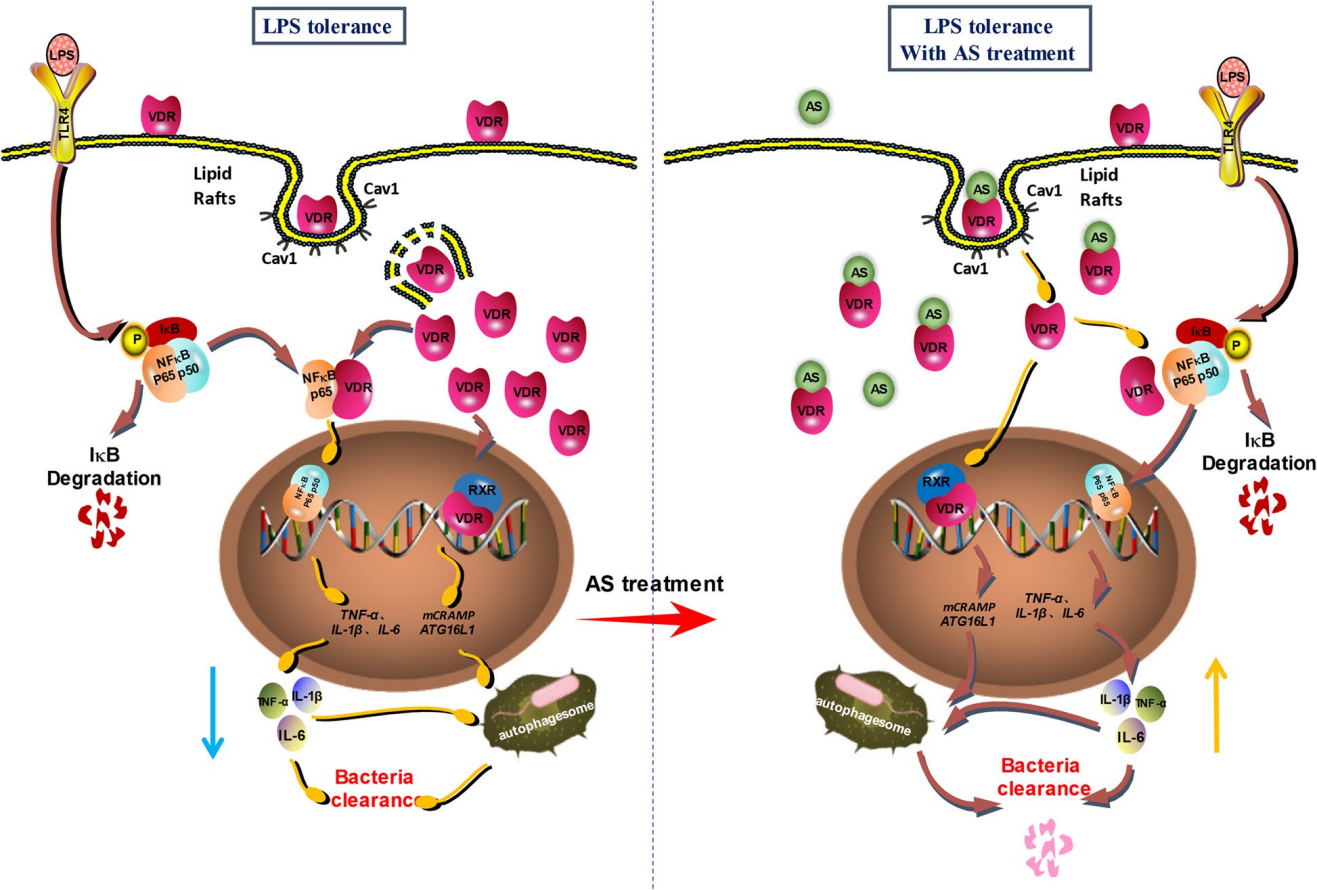
Schematic diagram of mVDR and its internalization within macrophages with or without artesunate treatment

Our previous results showed AS could inhibit cVDR’s translocation into the nucleus, leading to a significant reduction of nVDR in the nucleus [15]. Herein, we found that AS could reduce the fluorescence intensity of mVDR on cell membrane and significantly reduce the protein levels of VDR in the cell membrane, cytoplasm, and nucleus. Combined with previous experimental results, we speculated that the molecular mechanism of AS to reverse the formation of LPS tolerance might function in two ways: 1) By binding with mVDR, AS leads to the reduction of mVDR internalized into the cytoplasm, which further reduces cVDR translocation into the nucleus, ultimately leading to reduced levels and effects of nVDR; 2) decreased cVDR levels would lead to less NFκB p65 binding, thus leading to an increase of NFκB p65 nuclear translocation and upregulation of pro-inflammatory cytokines expression, ultimately increasing pro-inflammatory cytokines release and enhancing autophagy (Fig. 9).

In summary, mVDR exists on the macrophage membrane and might be internalized into the cytoplasm via the caveolin-dependent lipid raft pathway. By binding to mVDR, AS reduces the entry of mVDR into the cytoplasm, thereby reducing nuclear translocation of cVDR, and leading to a reduced nVDR level.

## Supplementary Information

**Additional file 1.**

## Data Availability

All data needed to evaluate the conclusions in the manuscript are present in the manuscript.
