# Supplementary material for: Vitamin D receptor (VDR) on the cell membrane of mouse macrophages participates in the formation of lipopolysaccharide tolerance: mVDR is related to the effect of artesunate to reverse LPS tolerance
Source: Cell Commun Signal. 2023 May 29;21:124. doi: 10.1186/s12964-023-01137-w (PMC10227983; doi:10.1186/s12964-023-01137-w)
Supplement: Supplementary file 2 — Additional file 1. [file 12964_2023_1137_MOESM1_ESM.zip › Original blot images Material 2.pdf]

Fig.2 B1

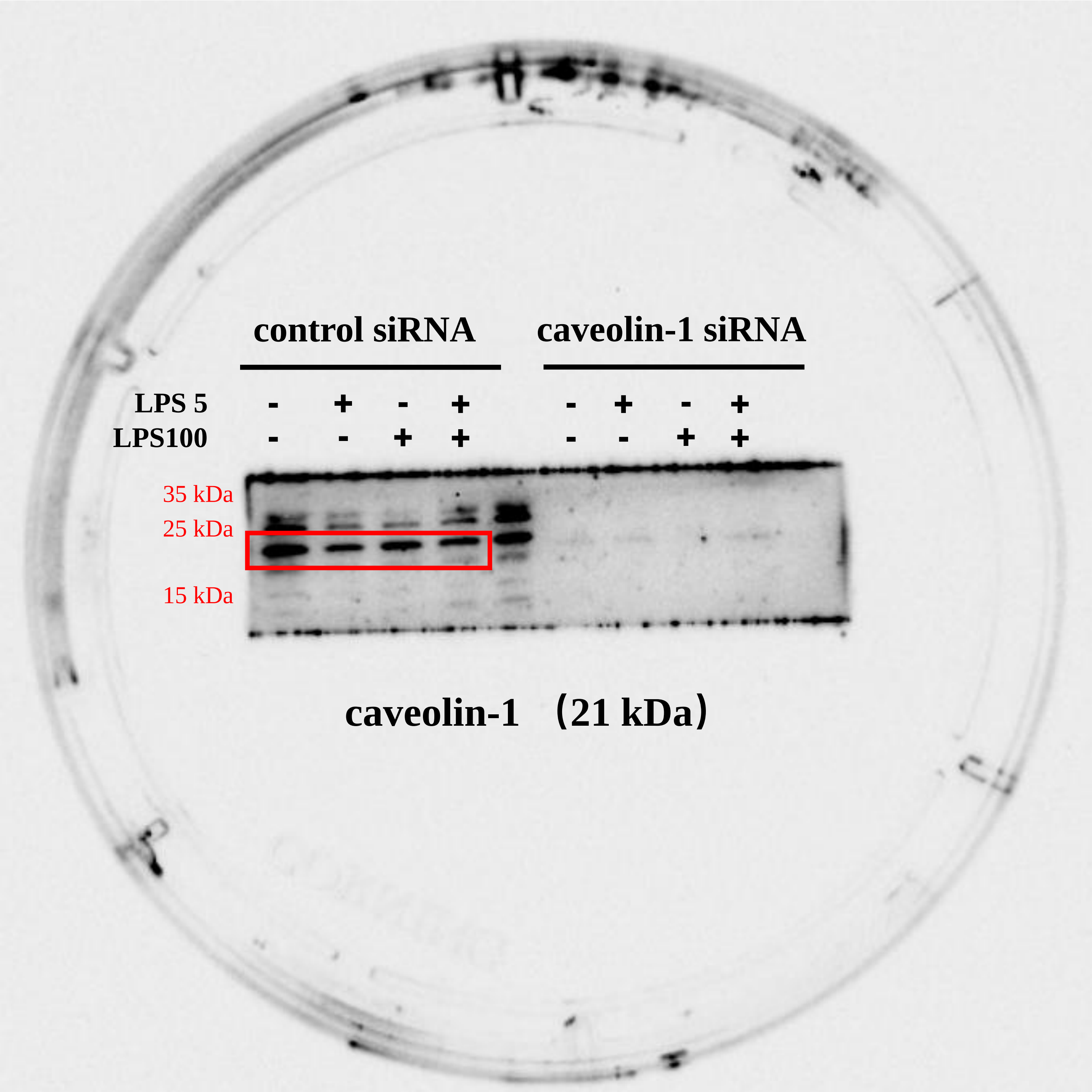
control siRNA
caveolin-1 siRNA
LPS 5 - + - + - + - +
LPS100 - - + + - - + +
35 kDa
25 kDa
15 kDa
caveolin-1 （21 kDa）

Fig.2 B1

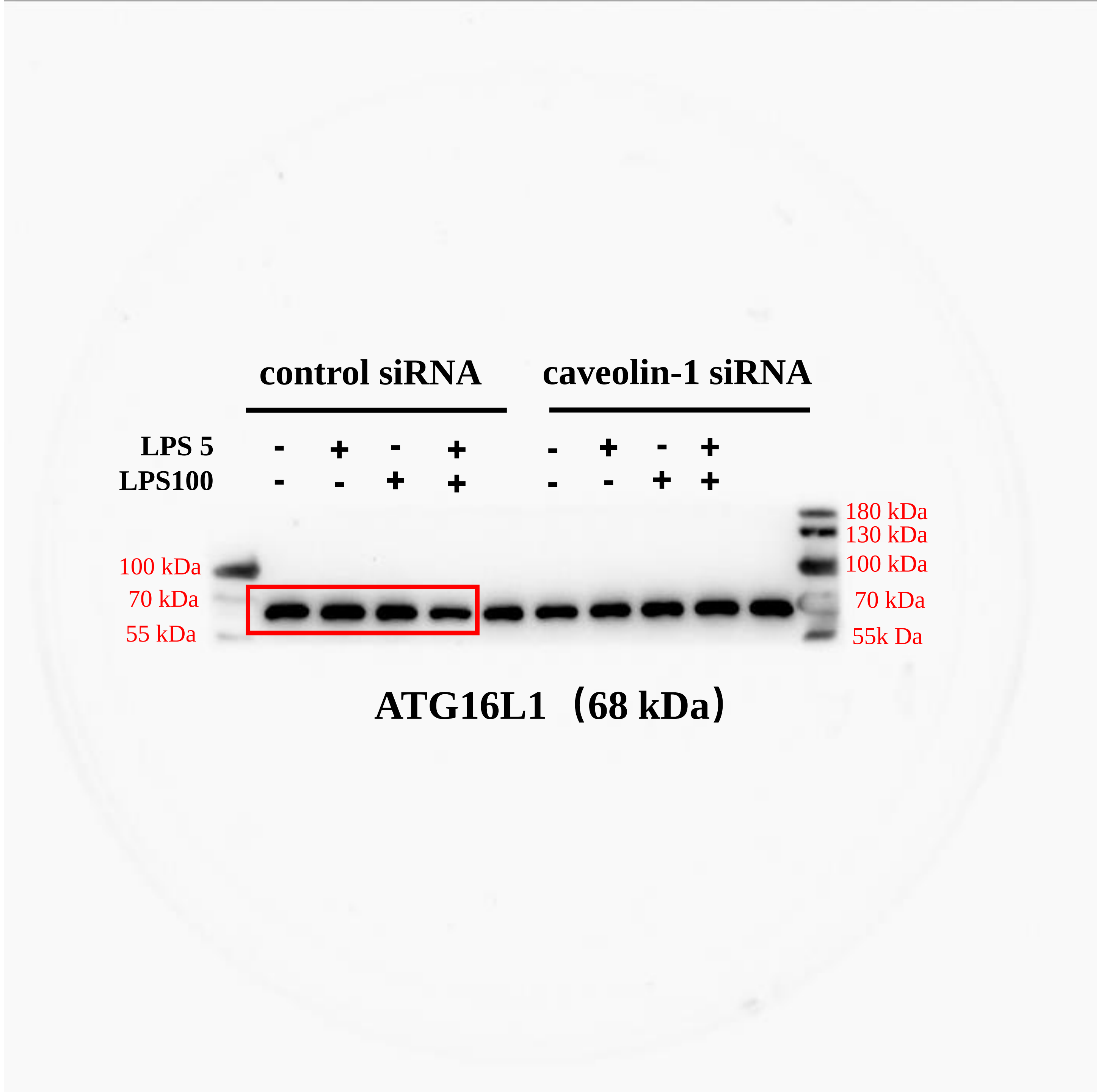

control siRNA
caveolin-1 siRNA
LPS 5
LPS100
- + - +
- - + +
- + - +
- - + +
100 kDa
70 kDa
55 kDa
180 kDa
130 kDa
100 kDa
70 kDa
55k Da
ATG16L1（68 kDa）

Fig.2 B1

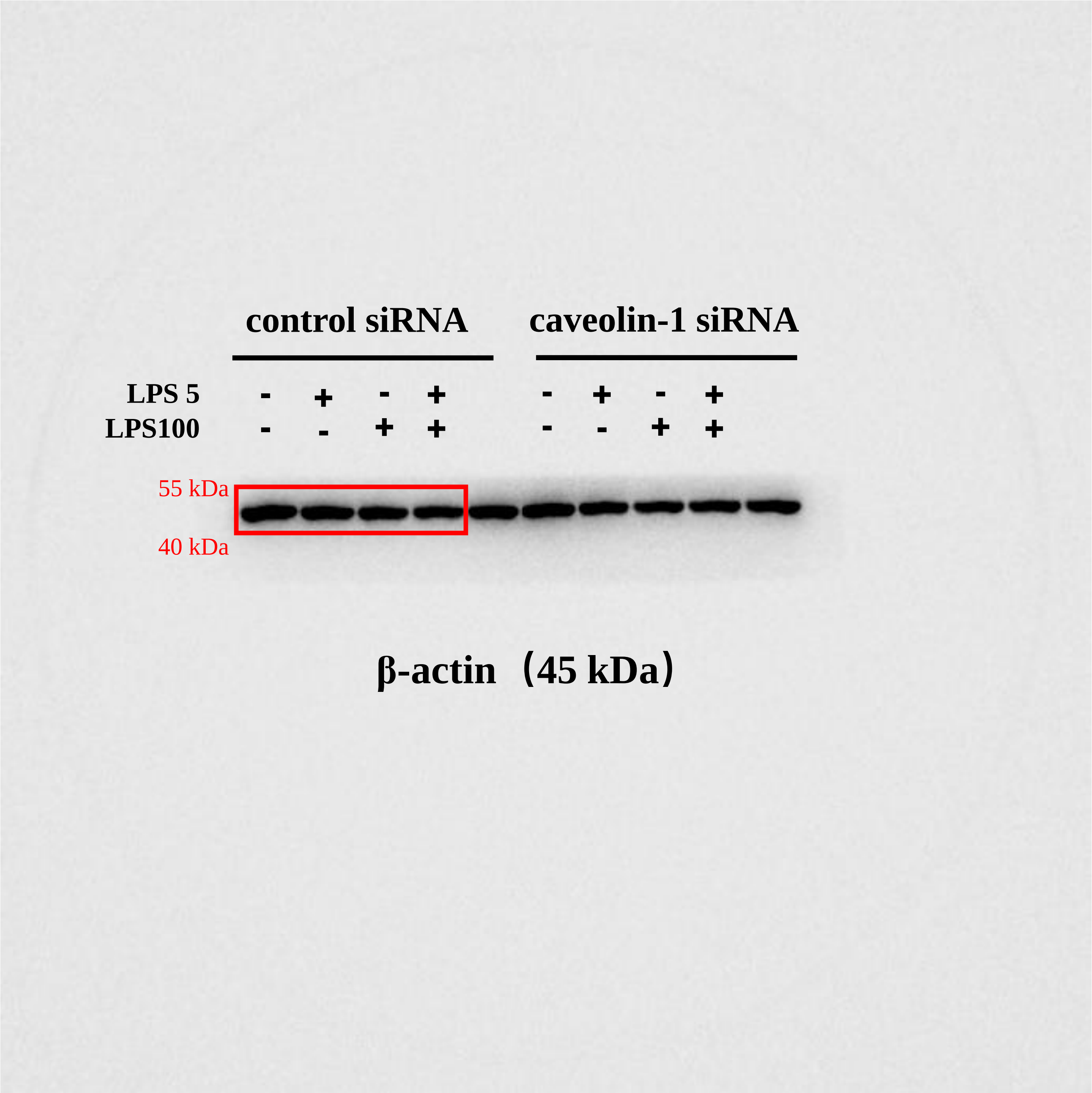
control siRNA
caveolin-1 siRNA
LPS 5
LPS100
- + - + - + - +
- - + + - - + +
55 kDa
40 kDa
β-actin（45 kDa）

Fig.2 B1

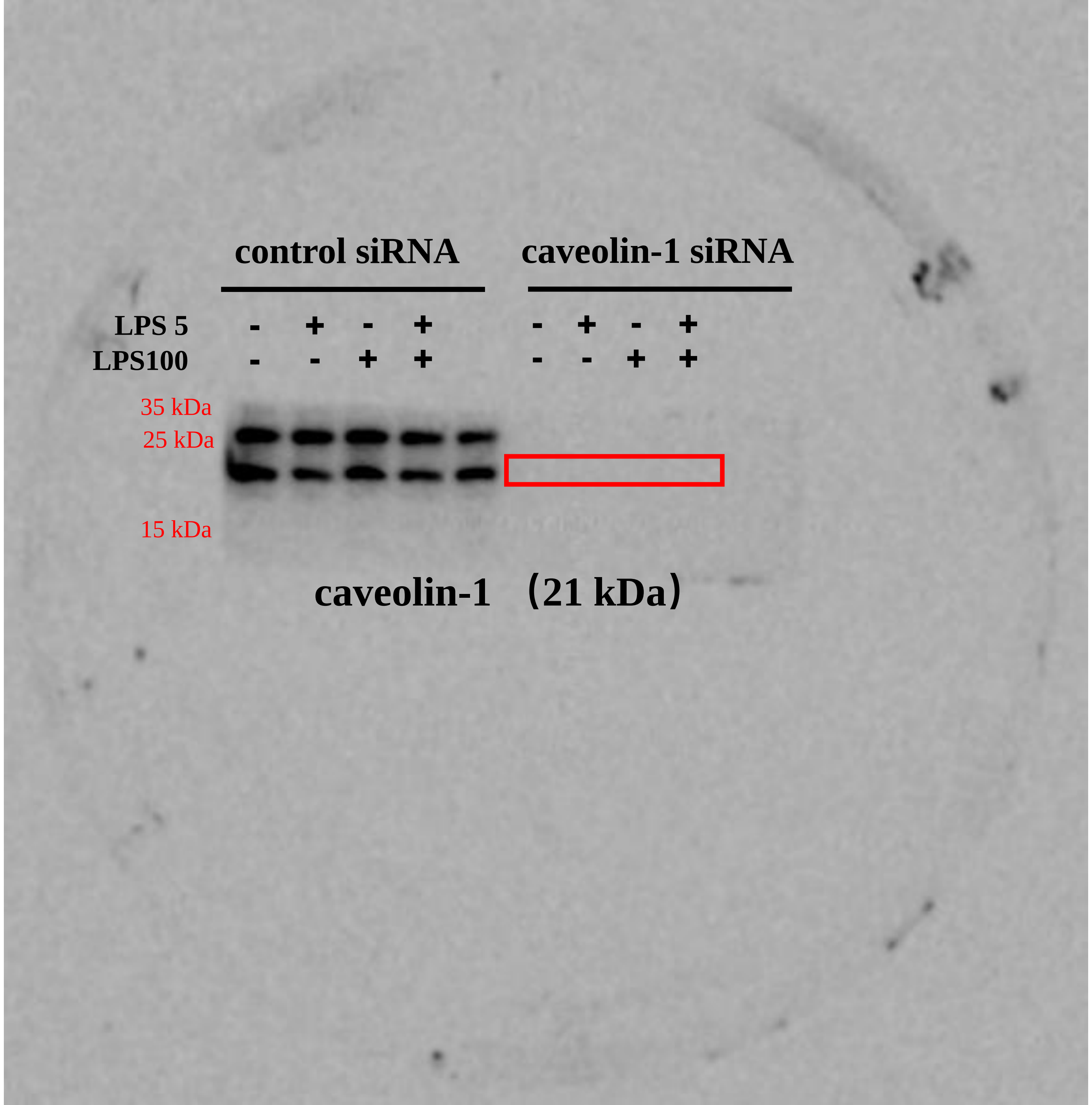
control siRNA
caveolin-1 siRNA
LPS 5 - + - + - + - +
LPS100 - - + + - - + +
35 kDa
25 kDa
15 kDa
caveolin-1 （21 kDa）

Fig.2 B1

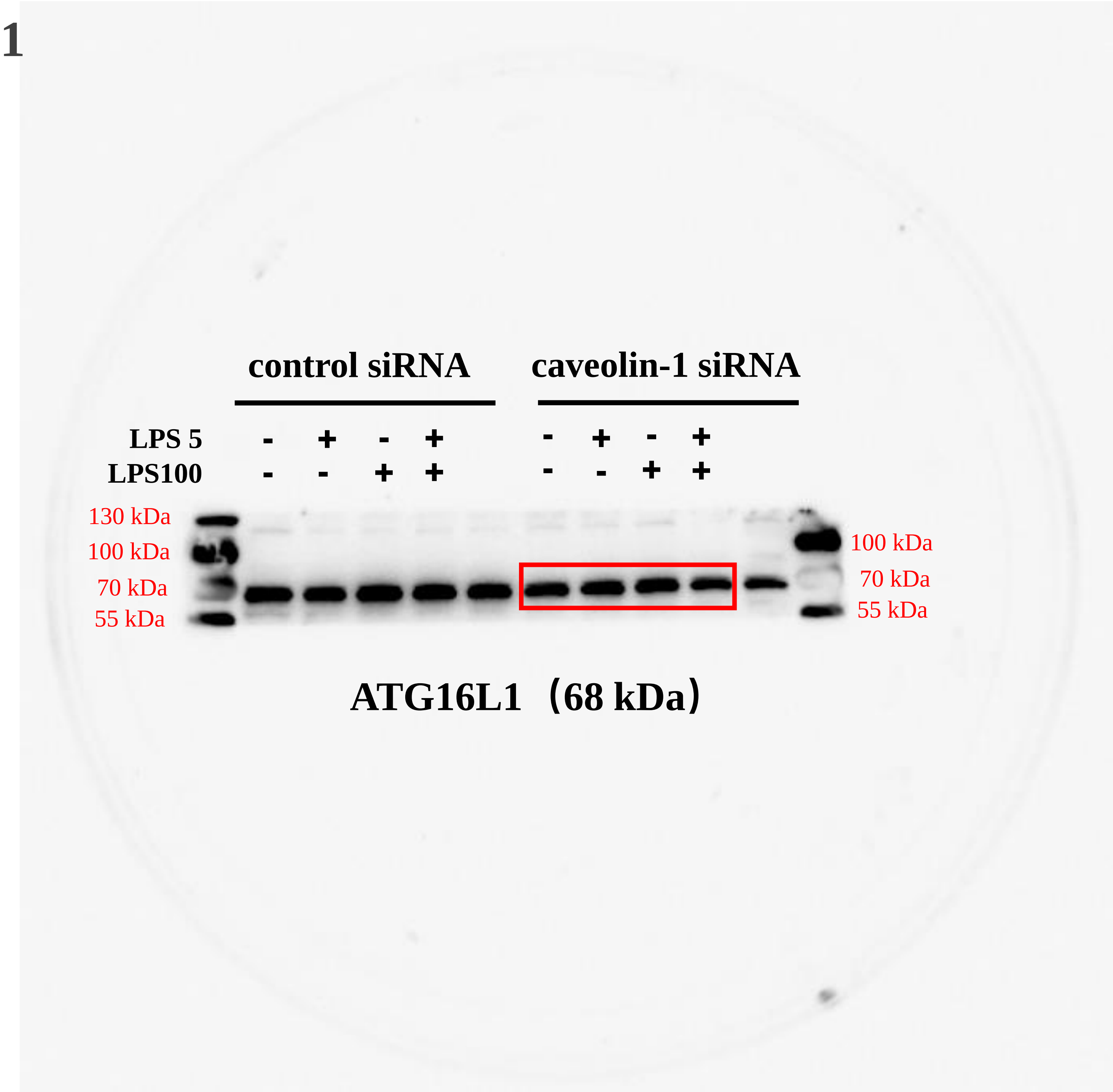
control siRNA
caveolin-1 siRNA
LPS 5
LPS100
130 kDa
100 kDa
70 kDa
55 kDa
100 kDa
70 kDa
55 kDa
ATG16L1（68 kDa）

Fig.2 B1

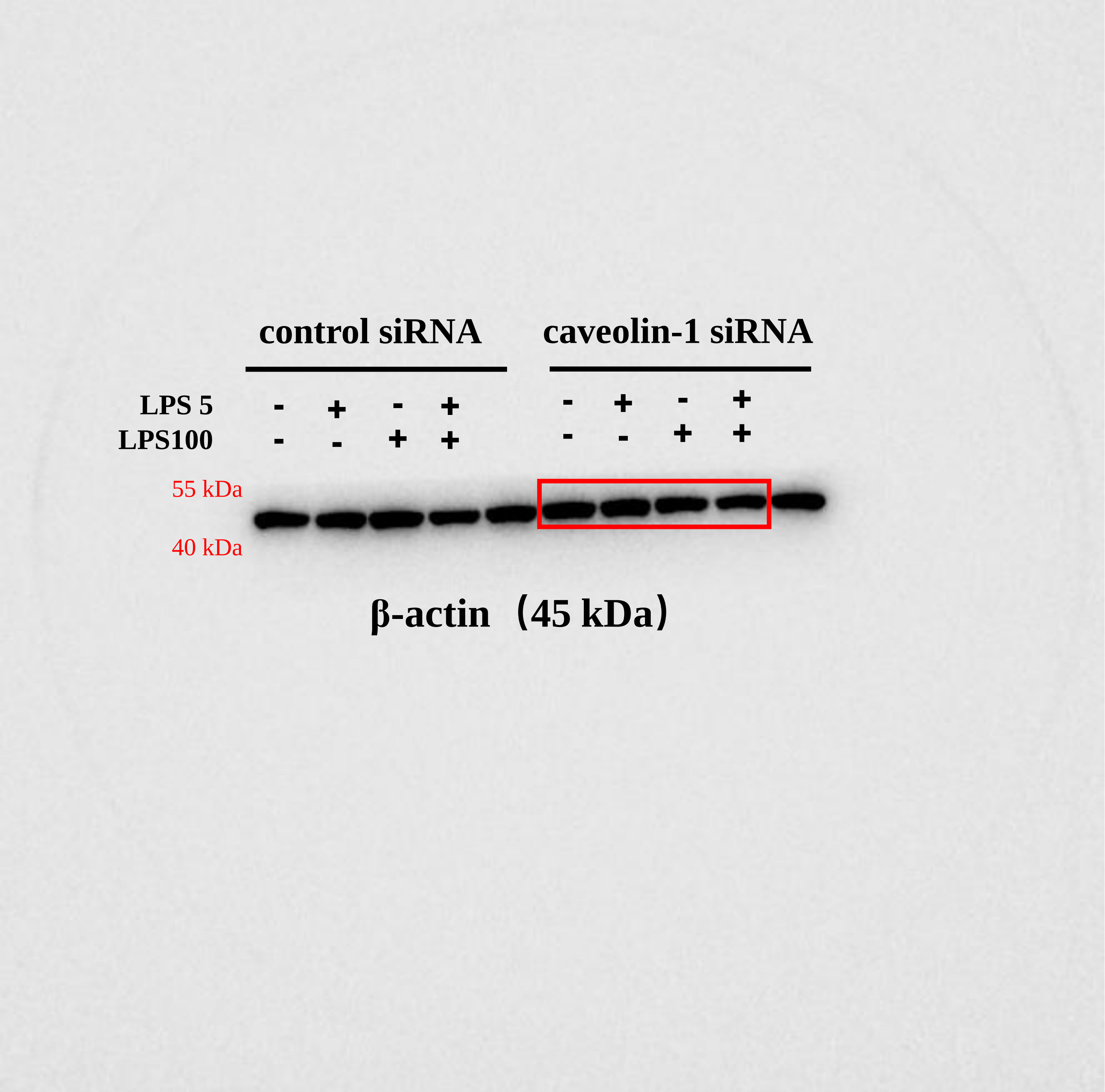
control siRNA
caveolin-1 siRNA
LPS 5
LPS100
55 kDa
40 kDa
β-actin（45 kDa）

Fig.5 C1

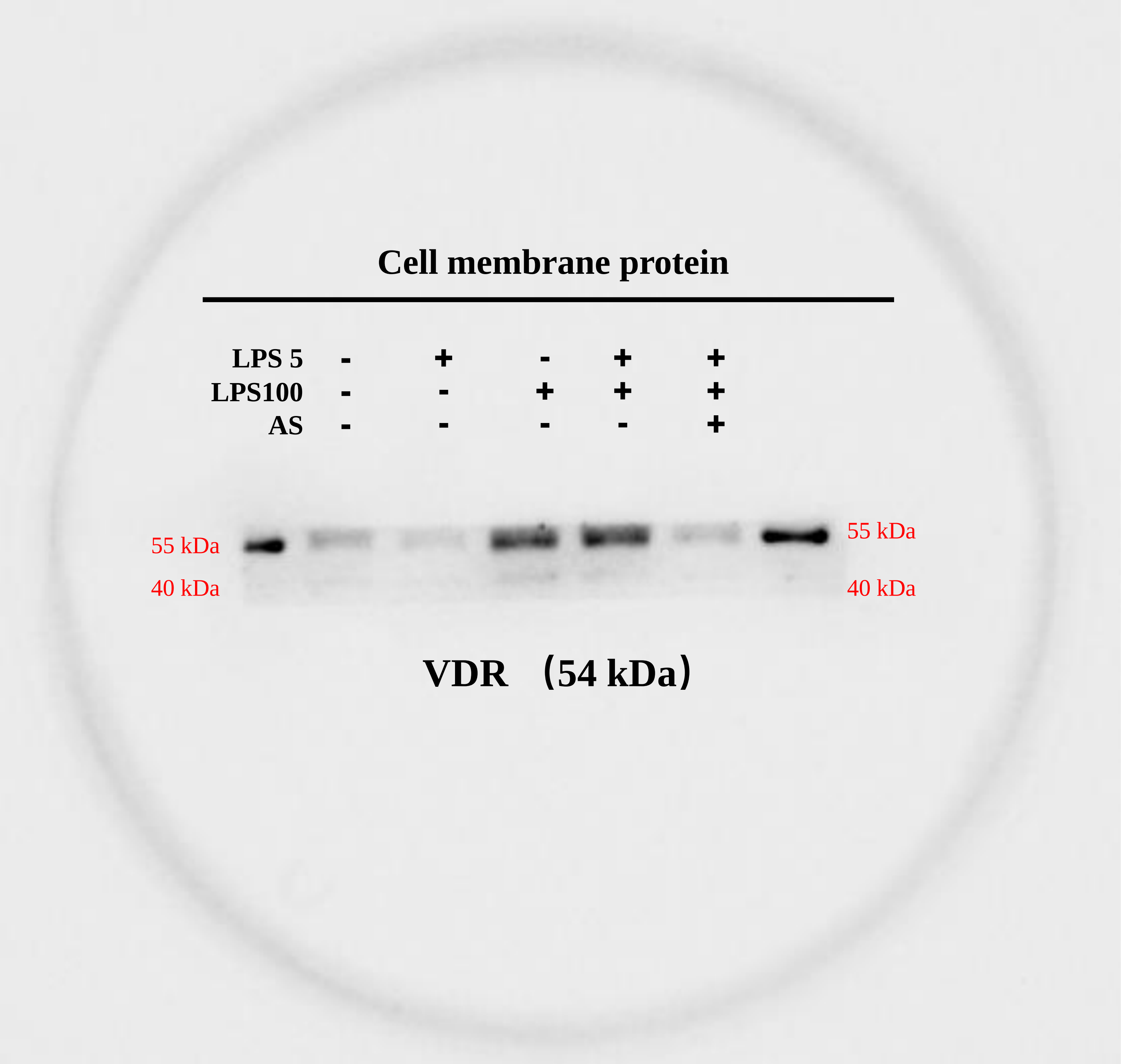
Cell membrane protein
LPS 5 - + - + +
LPS100 - - + + +
AS - - - - +
55 kDa
40 kDa
55 kDa
40 kDa
VDR （54 kDa）

Fig.5 C1

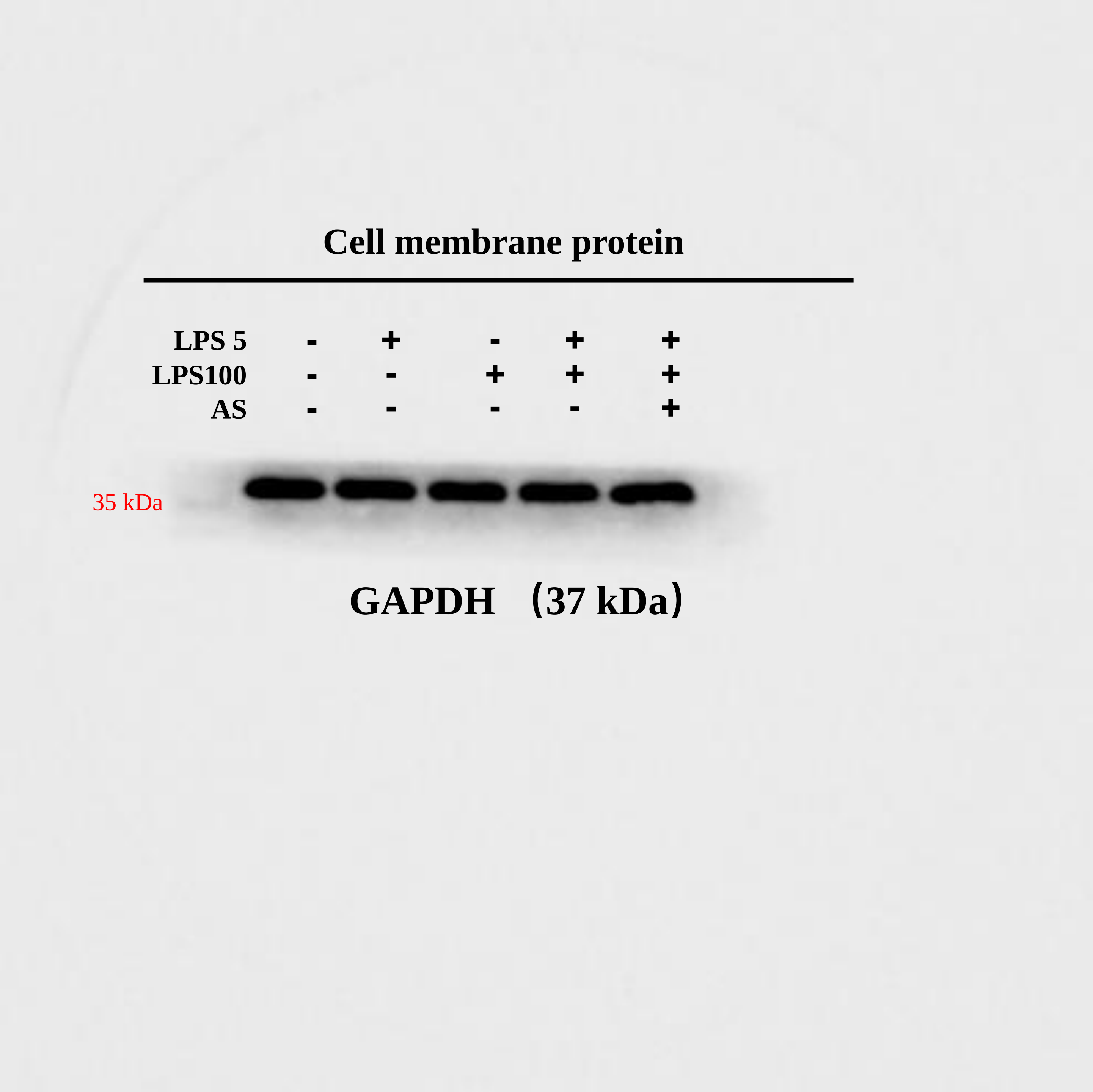

Fig.5 C2

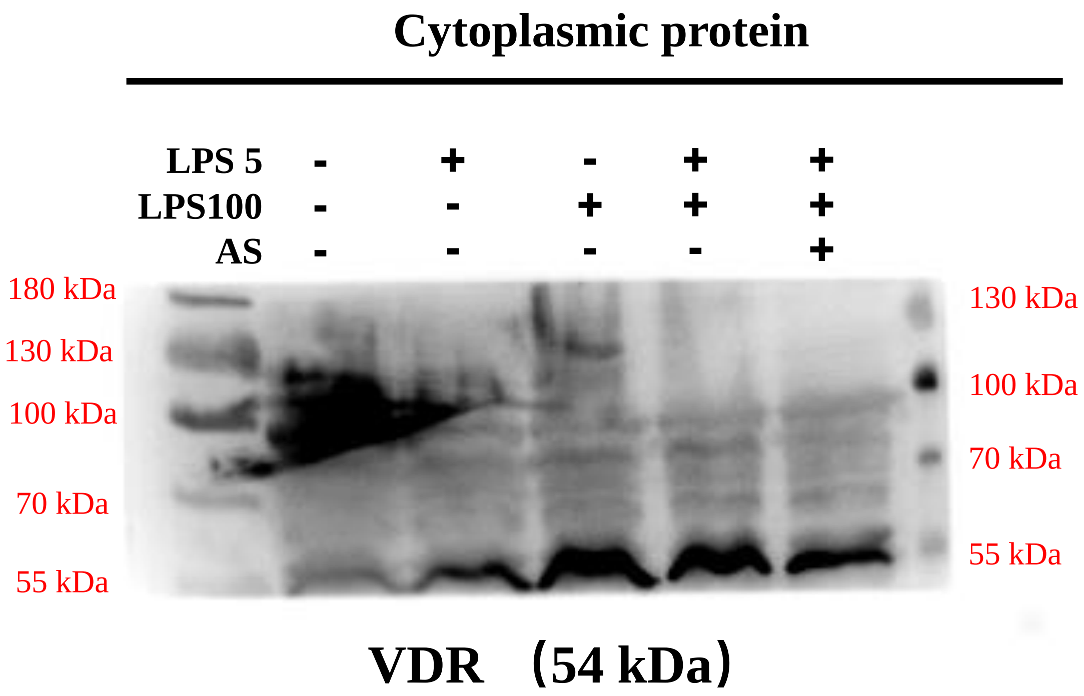
Cytoplasmic protein
LPS 5 - + - + +
LPS100 - - + + +
AS - - - - +
180 kDa
130 kDa
100 kDa
70 kDa
55 kDa
130 kDa
100 kDa
70 kDa
55 kDa
VDR （54 kDa）

Fig.5 C2

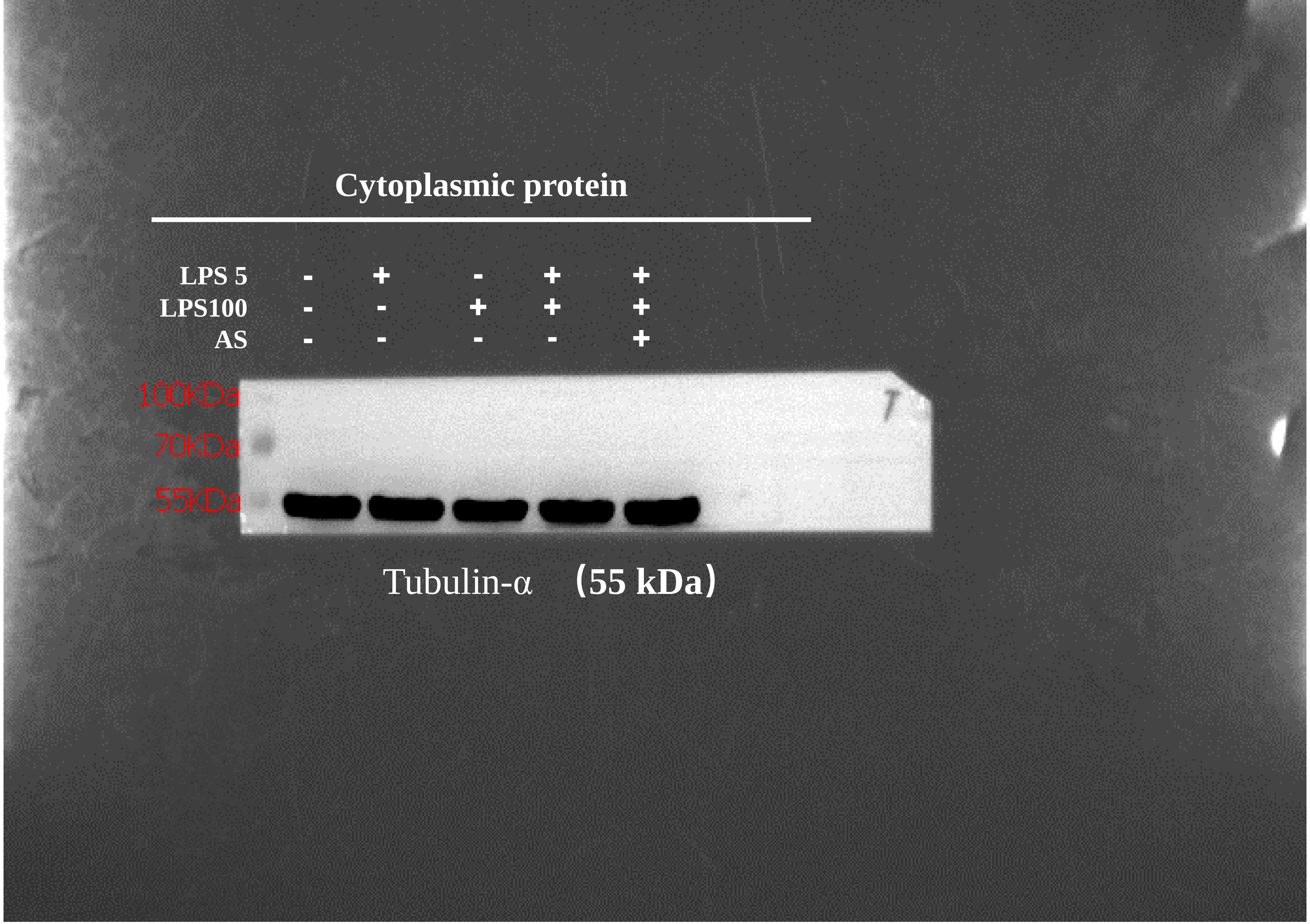
Cytoplasmic protein
LPS 5 - + - + +
LPS100 - - + + +
AS - - - - +
100KDa
70KDa
55KDa
Tubulin-α (55 kDa)

Fig.5 C3

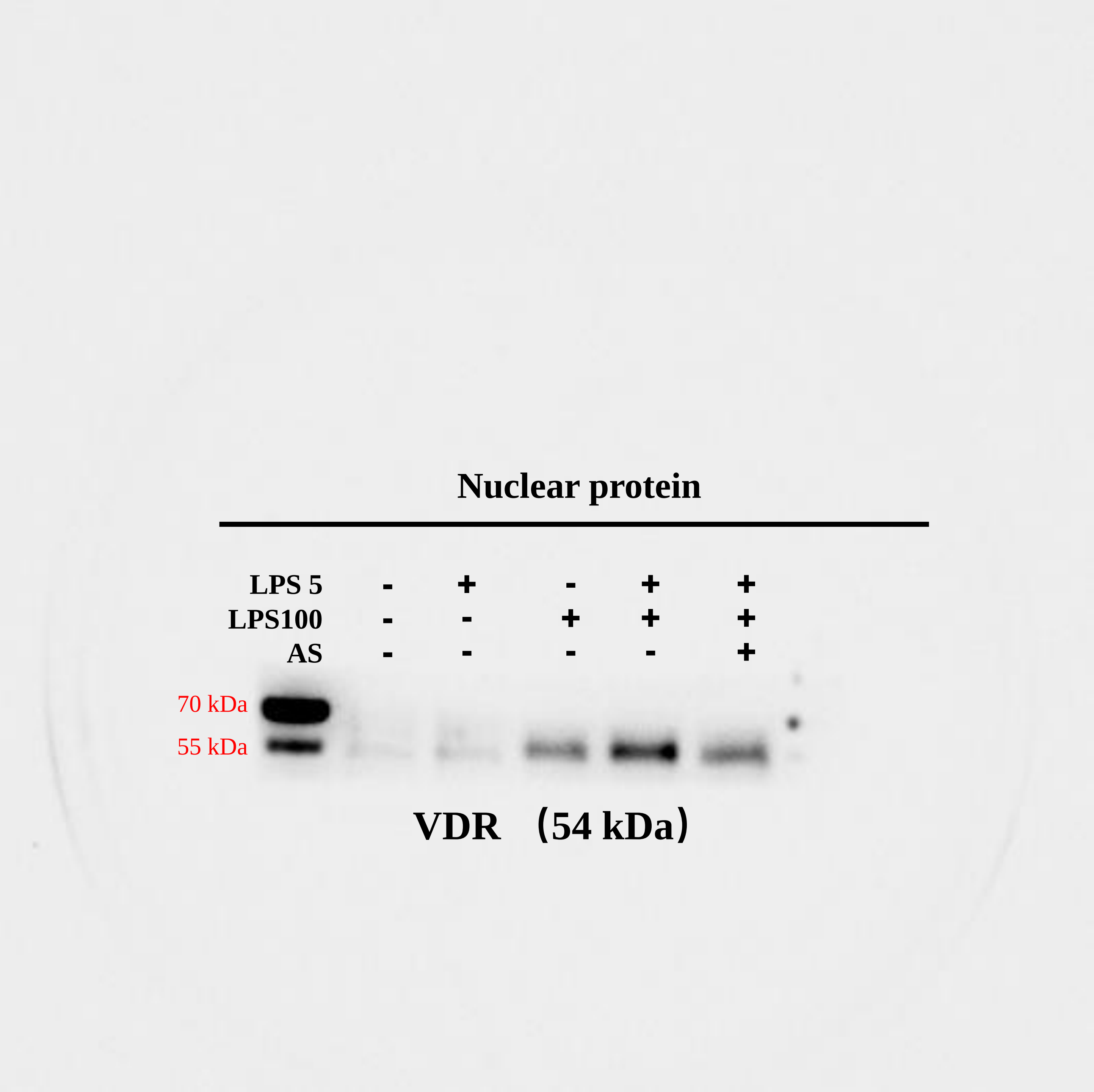

Fig.5 C3

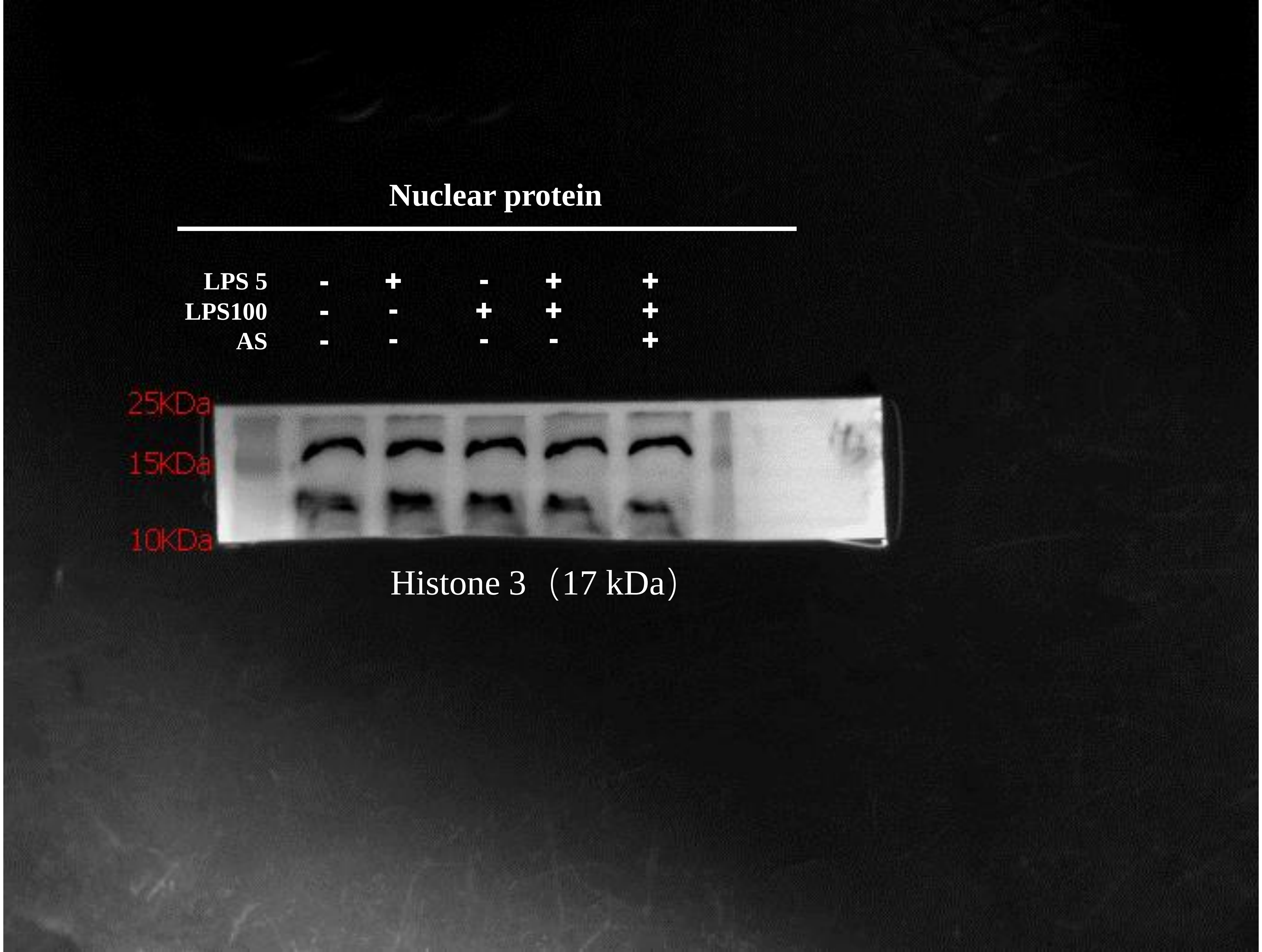
Nuclear protein
LPS 5 - + - + +
LPS100 - - + + +
AS - - - - +
25KDa
15KDa
10KDa
Histone 3（17 kDa）
